# Supplementary material for: Past Connectivity but Recent Inbreeding in Cross River Gorillas Determined Using Whole Genomes from Single Hairs
Source: Genes (Basel). 2023 Mar 18;14(3):743. doi: 10.3390/genes14030743 (PMC10048488; doi:10.3390/genes14030743)
Supplement: Supplementary file 1 [file genes-14-00743-s001.zip › SupplementaryMaterial.pdf]

Supplementary Figures

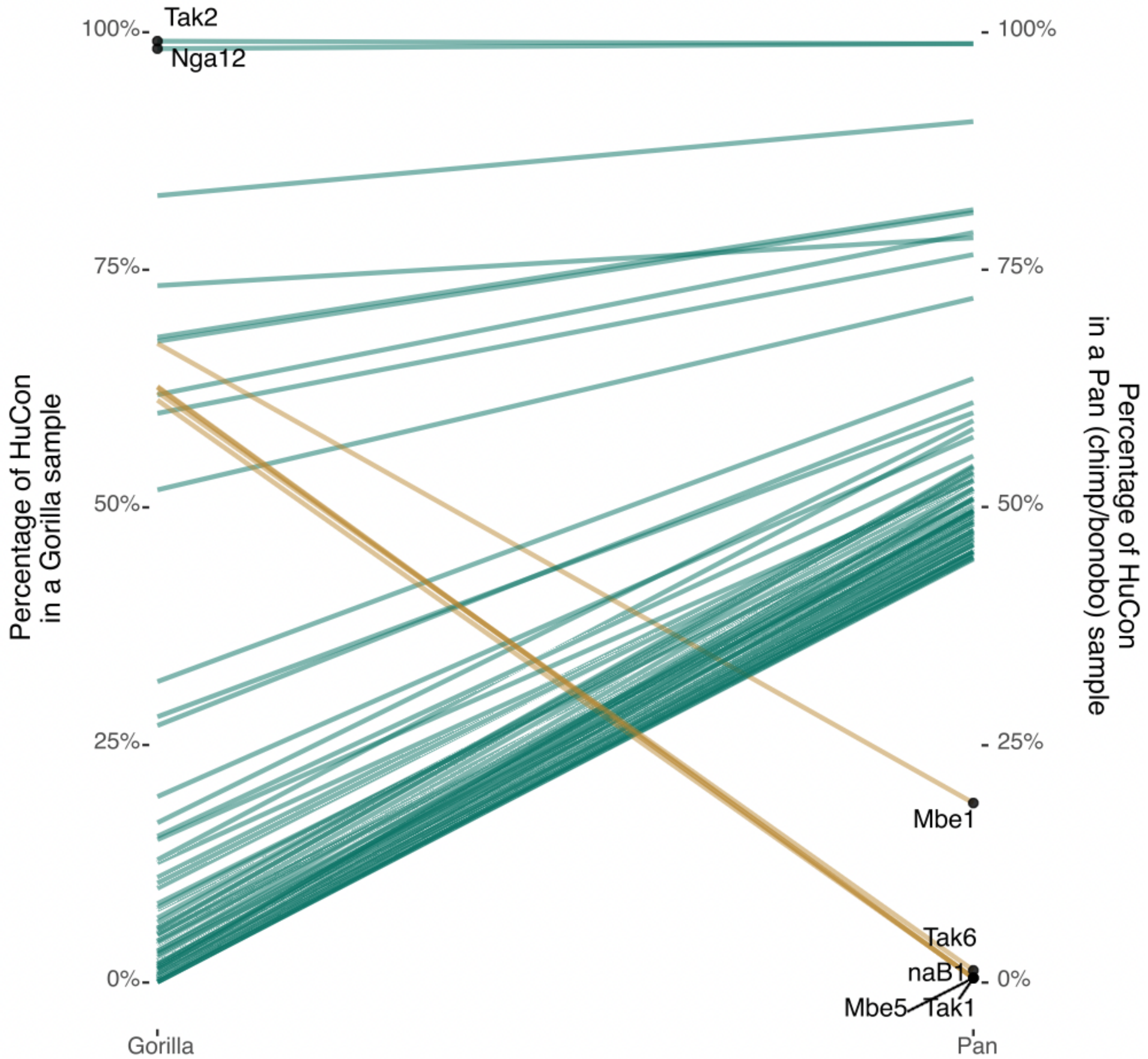

Figure S1. Great ape contamination test (HuConTest). HuConTest results with two different references, gorilla and pan (chimpanzee and bonobo). Gorilla hair samples are identified in green: when using gorilla they have a low percentage of HuCon (Human Contamination) and this estimation increases when setting another species reference (*pan*). Those samples that belong to chimpanzees, identified in yellow, show the opposite trend, with high levels of HuCon when using the gorilla as a reference and lower levels when using *pan*.

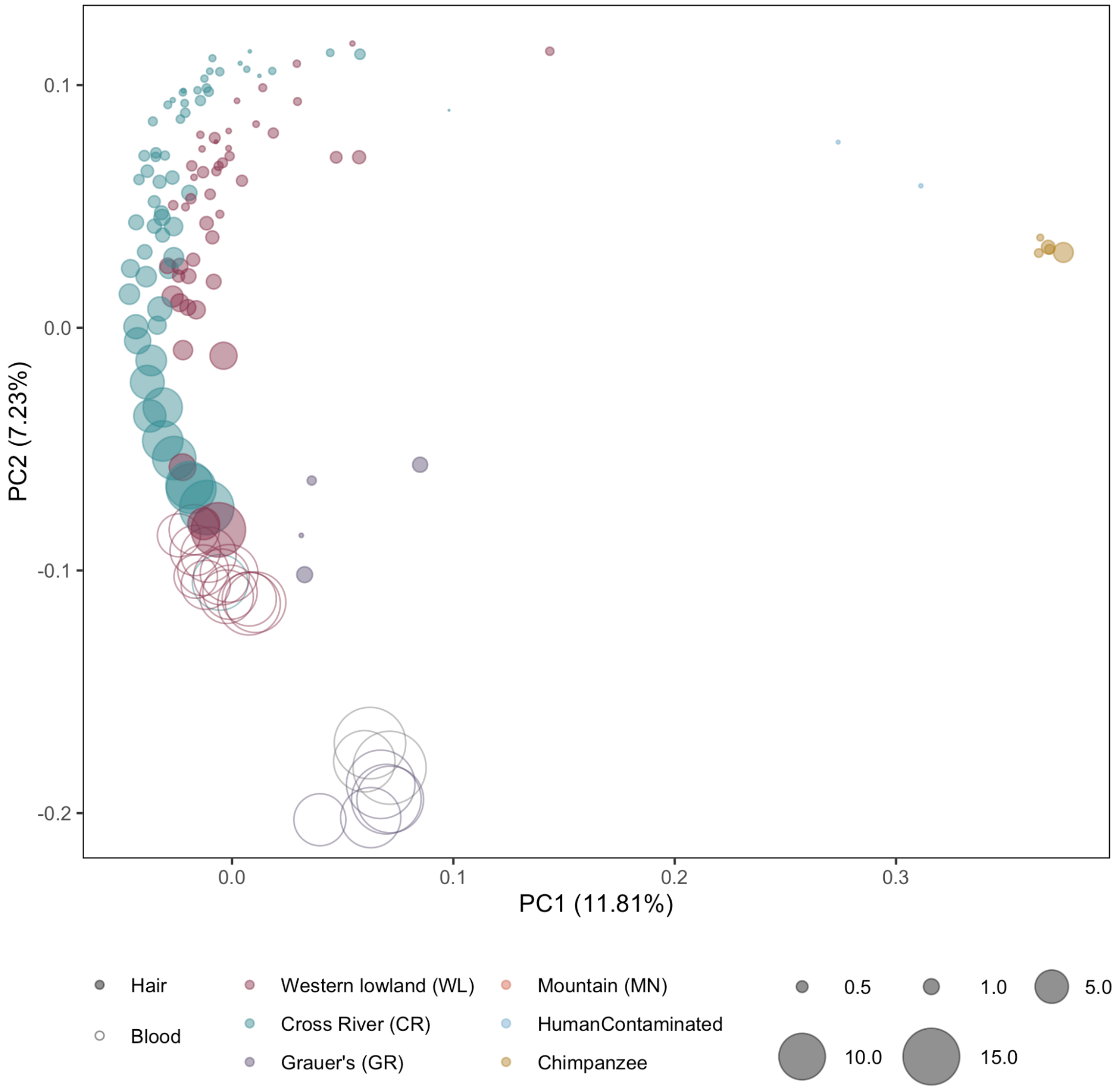

Figure S2. PCA without QC. PCA from PCANGSD using all the samples without quality-control filtering (Set01). Shapes represent the type of sample, hairs with filled circles and blood with empty circles. Color represents the gorilla subspecies, as well as identifies those samples that belong to chimpanzees in yellow, and those with >99% human contamination (from HuConTest) in light blue). Circle size represents the average coverage of each of the libraries. One can observe that PC1 is driven by the species, with chimpanzee samples pulling apart from gorilla samples. PC2 is mainly driven by coverage, as indicated by circle size.

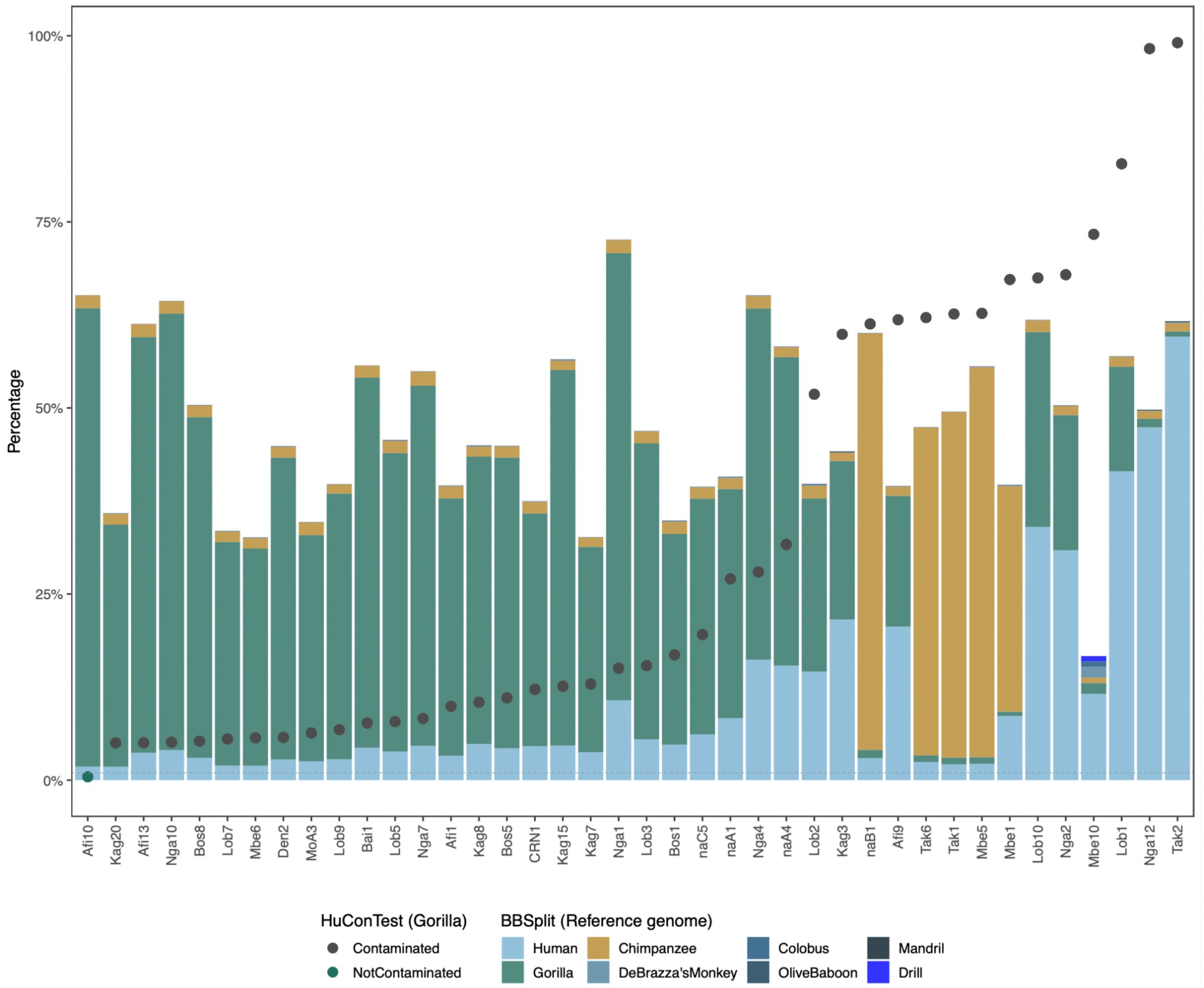

Figure S3. Contamination test. Bars represent BBsplit results on those samples with HuCon percentage above 5% plus one sample with HuCon <1%. BBSplit was run using the following references: human, gorilla, chimpanzee, de Brazza's monkey, colobus, olive baboon, mandrill, and drill as possible species. Dots represent HuConTest results for these samples.

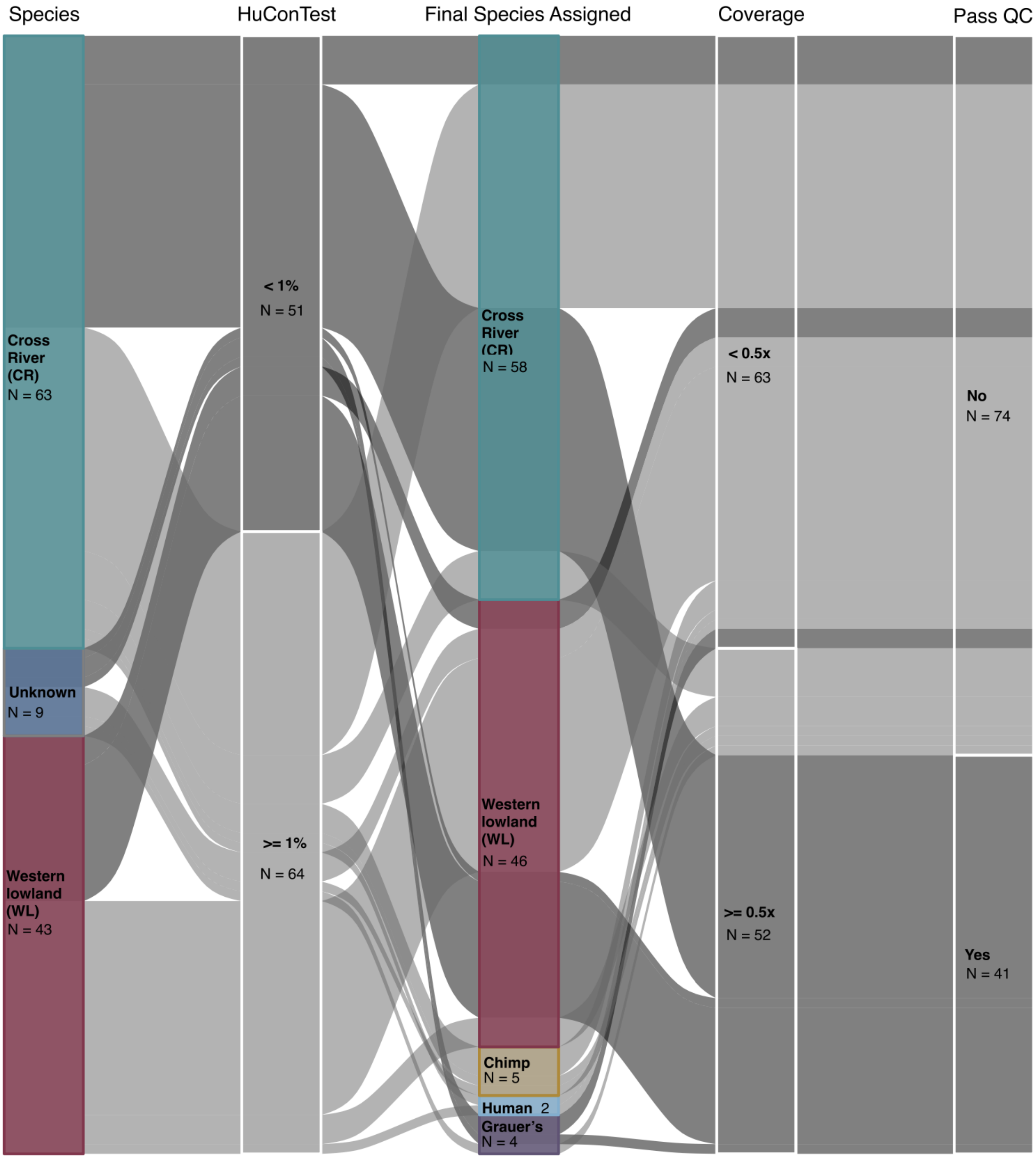

Figure S4. Quality control. The first column represents the counts of the number of samples in the dataset at the beginning of the project and their putative species: Cross River gorilla, western lowland gorilla and some samples had unknown origin. Then, from the results of the HuConTest, libraries were labelled as Yes or No, depending on if they had a HuConTest value above 1% (which we considered as a putatively-contaminated sample). Using both the HuConTest and BBSplit we were able to identify two samples with high percentage of human contamination (HuConTest > 99%) and 5 chimpanzee samples (see Supplementary Figures 1-2). We also identified some samples belonging to Grauer's gorilla subspecies after principal component analysis (see Supplementary Figure 6). Finally, we also determine a minimum of 0.5x of coverage as the threshold to consider a sample for further analyses (fourth column, below 0.5x or above and equal to 0.5x). The fifth column shows the amount of samples that pass all the quality controls (HuConTest < 1% and average depth > 0.5x).

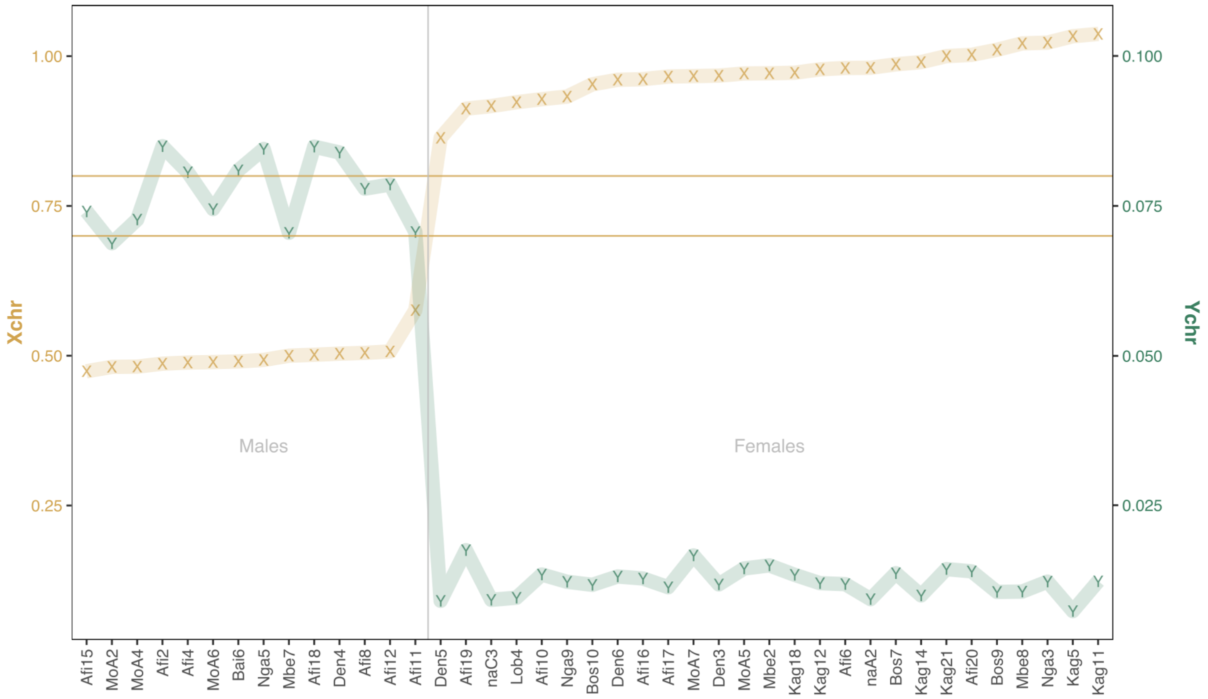

Figure S5. Sex determination from coverage. Sex chromosome coverage ratio versus average autosomal coverage, X_{chr}:A_{chr} and Y_{chr}:A_{chr}. Y chromosome coverage ratio is indicated in green and X chromosome coverage ratio in yellow. Those samples that belong to males (XY) will have a higher Y_{chr} ratio, while female samples (XX) will have an X_{chr} ratio above 0.8x and very small Y_{chr} ratios.

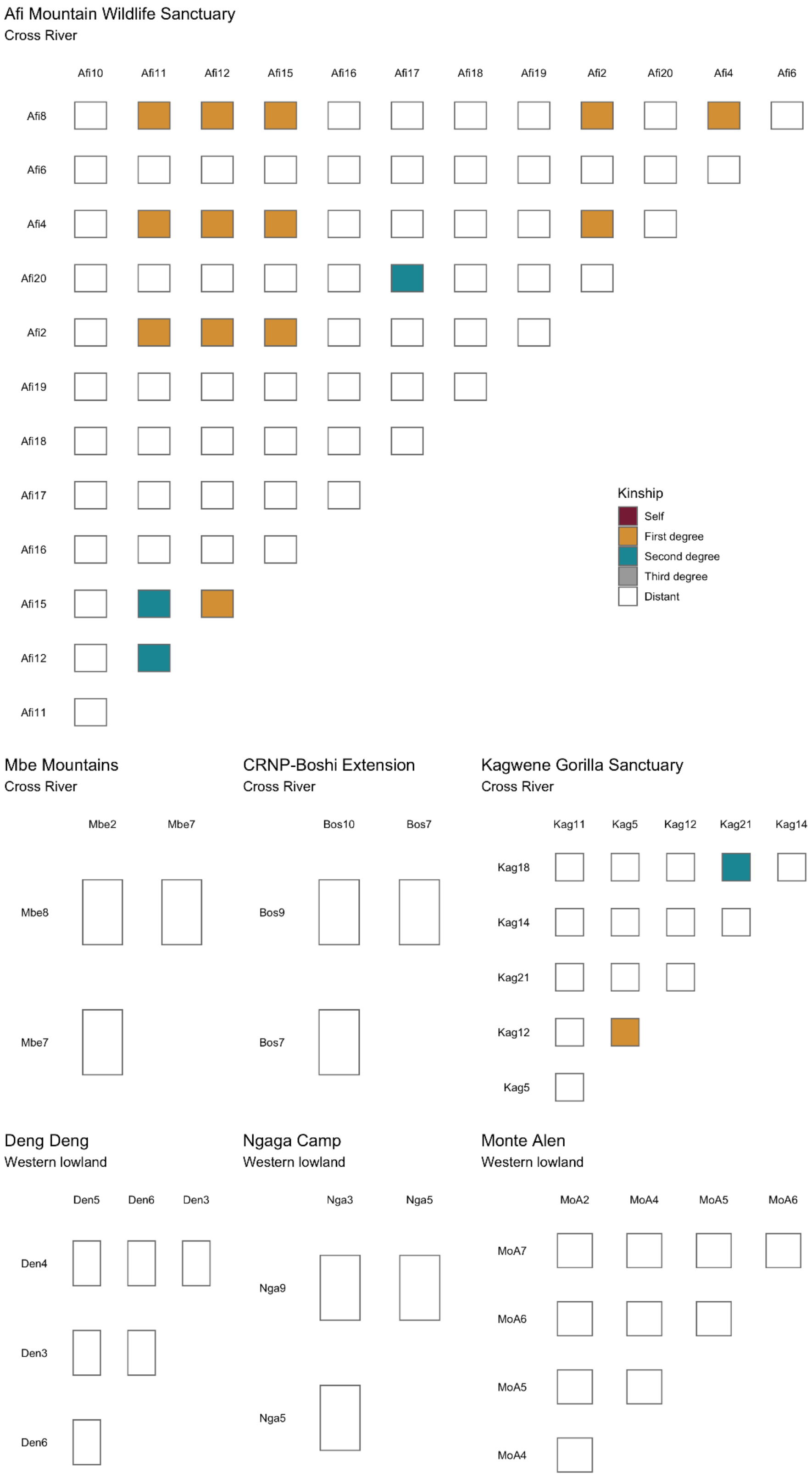

Figure S6. Relatedness. NGSRelateV2 results per site. Cell color indicates the kinship level (self, first degree, second degree, third degree or distant).

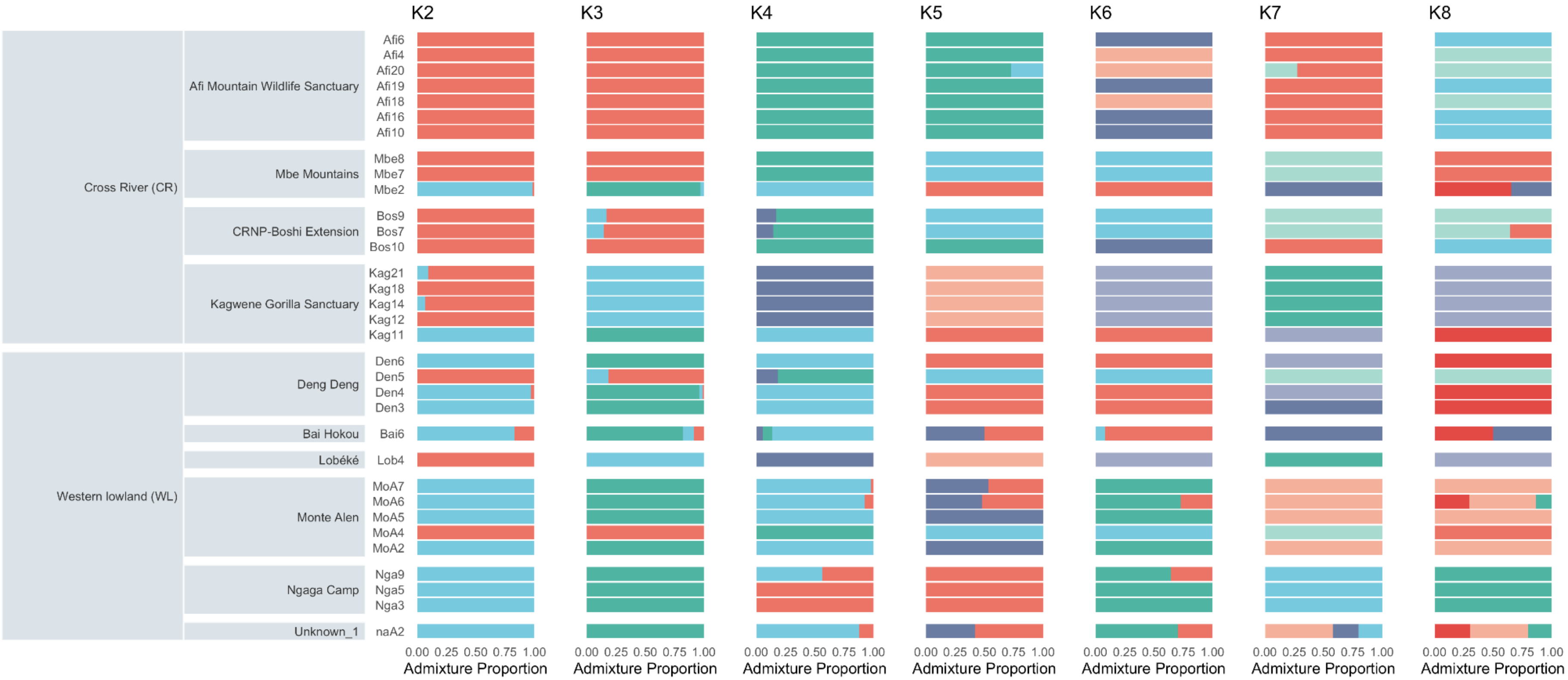

Figure S7. Admixture western gorillas. NGSadmix results using western gorilla (WL and CR) hair samples after quality control (Set05). Colors indicate each component.

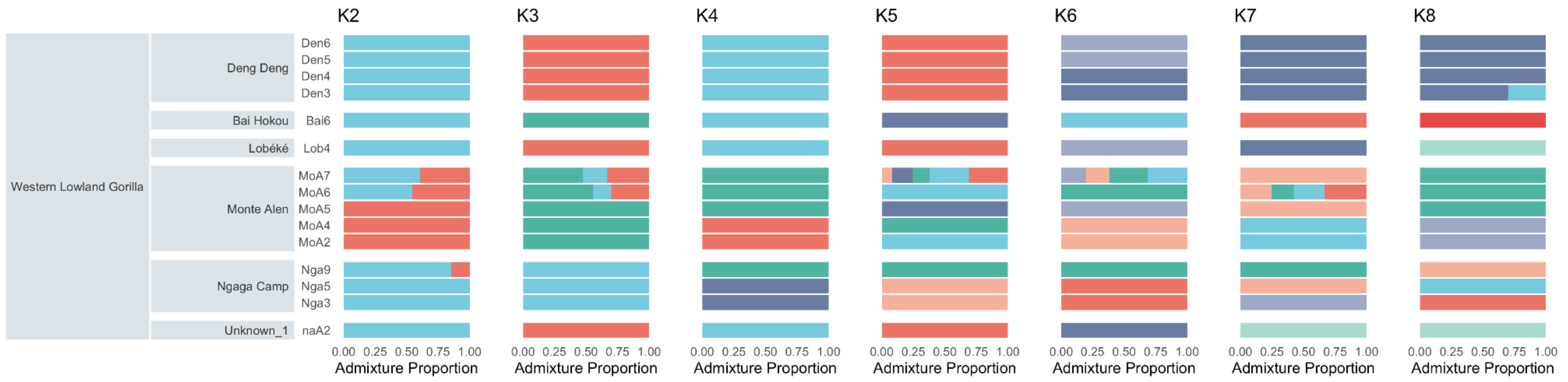

Figure S8. Admixture WL gorilla hairs. NGSadmix results using western lowland gorilla (WL) hair samples after quality control (Set06). Colors indicate each component.

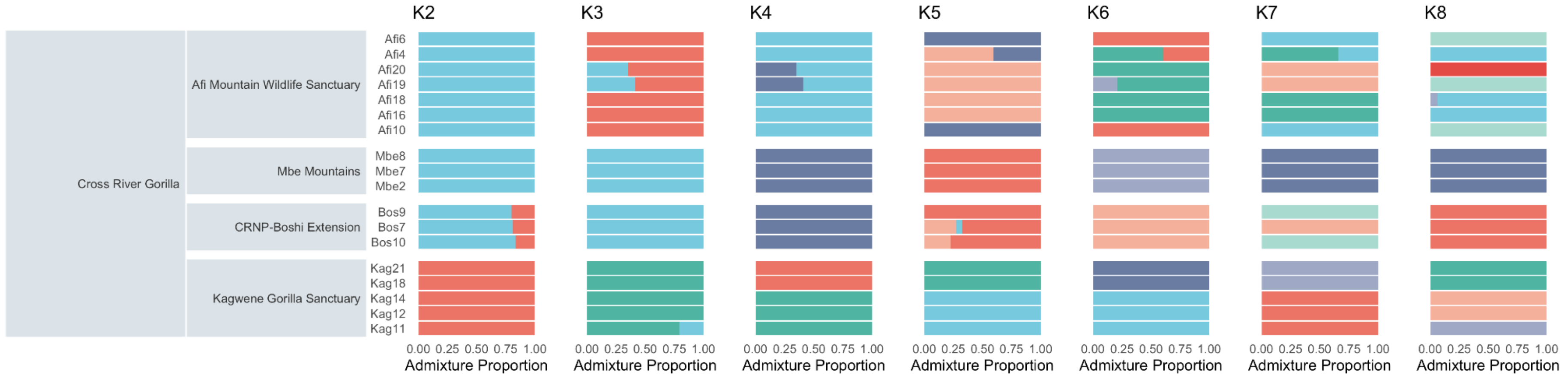

Figure S9. Admixture CR gorilla hairs. NGSadmix results using Cross River gorilla (CR) hair samples after quality control (Set07). Colors indicate each component.

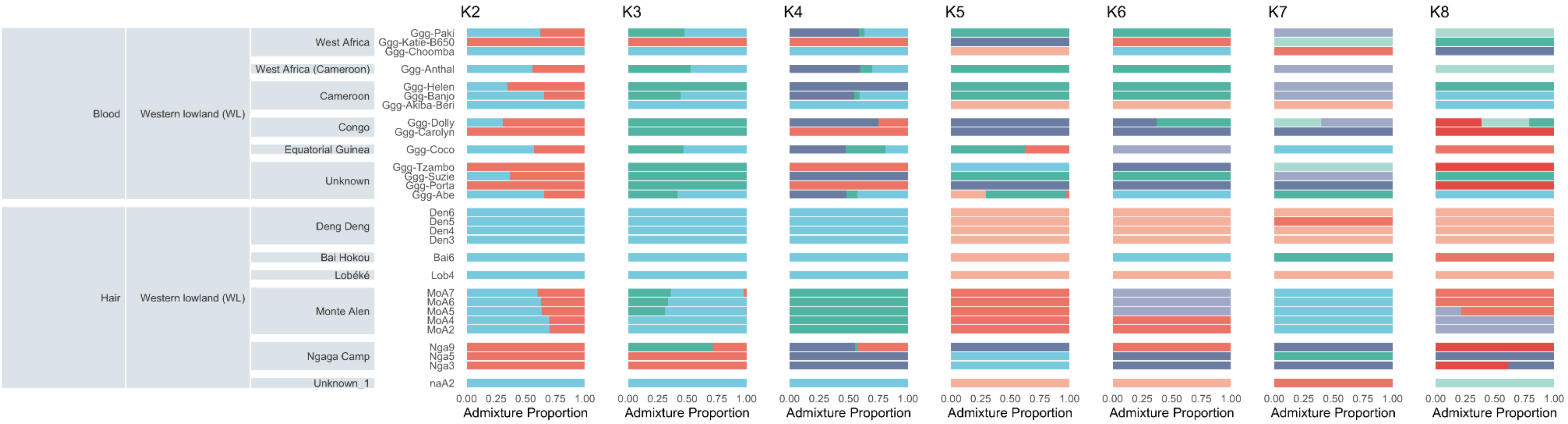

Figure S10. Admixture WL hairs and blood. NGSadmix results using western lowland gorilla (WL) hair and blood samples (Set03). Colors indicate each component.

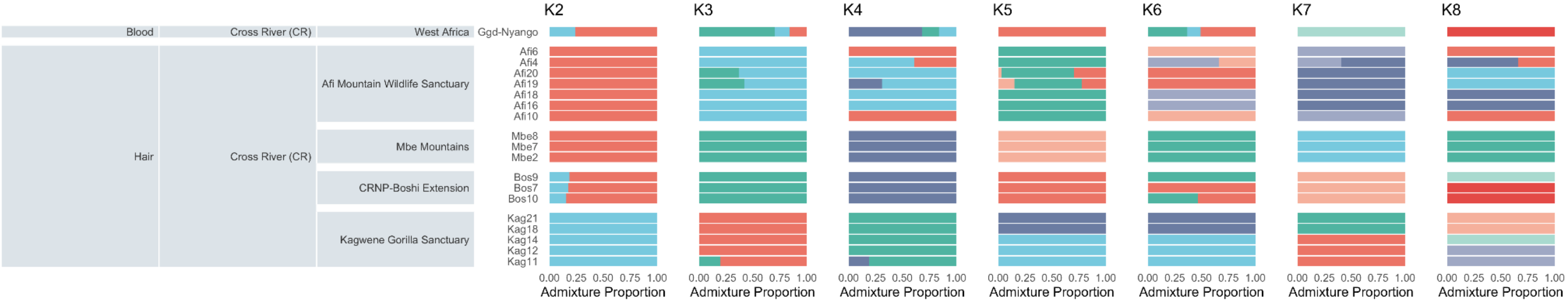

Figure S11. Admixture CR hairs and blood. NGSadmix results using Cross River gorilla (CR) hair and blood samples (Set04). Colors indicate each component.

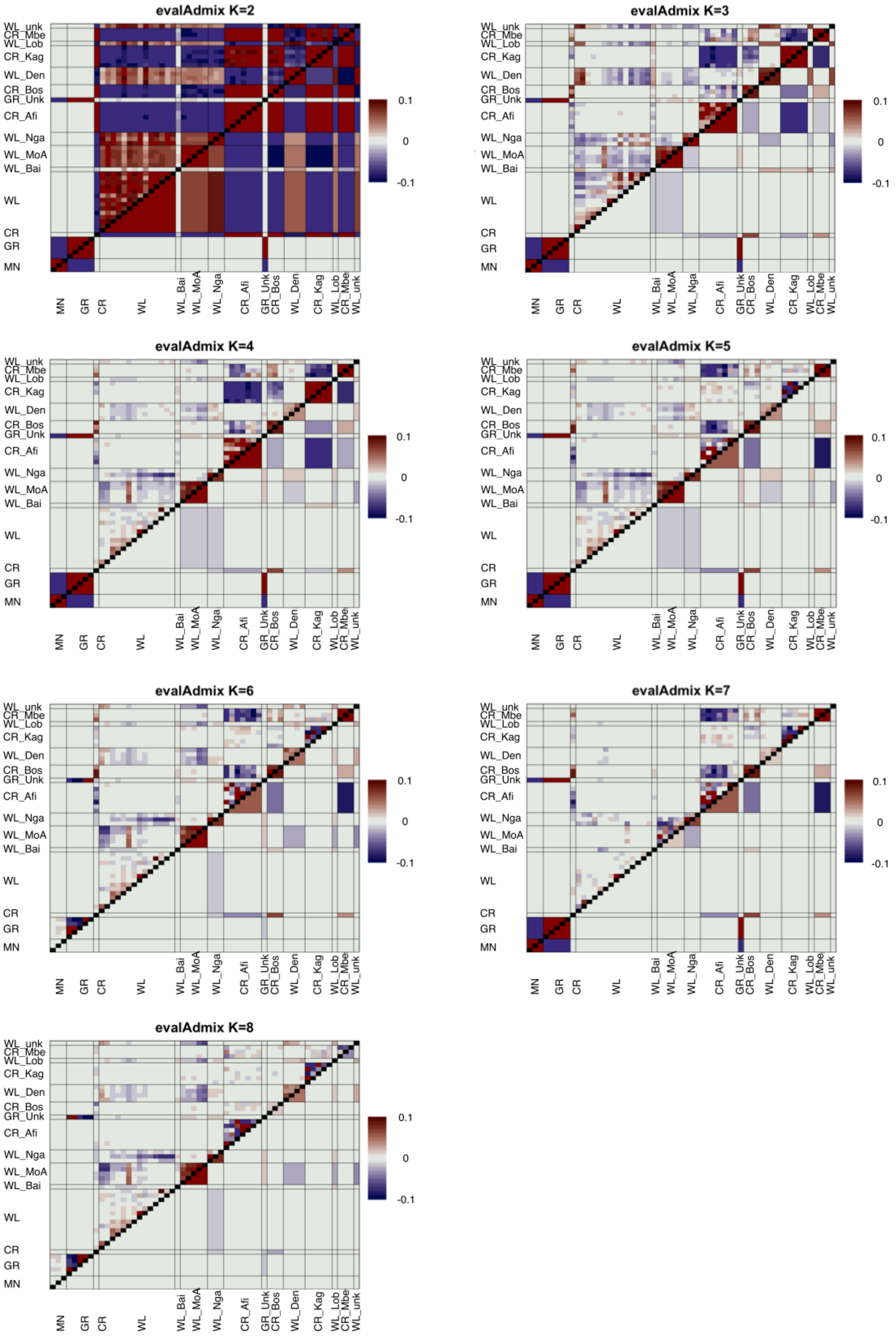

Figure S12. Evaladmix. Results from evalAdmix from K 2 to 8 on western gorilla species hairs (CR and WL) (Set05).

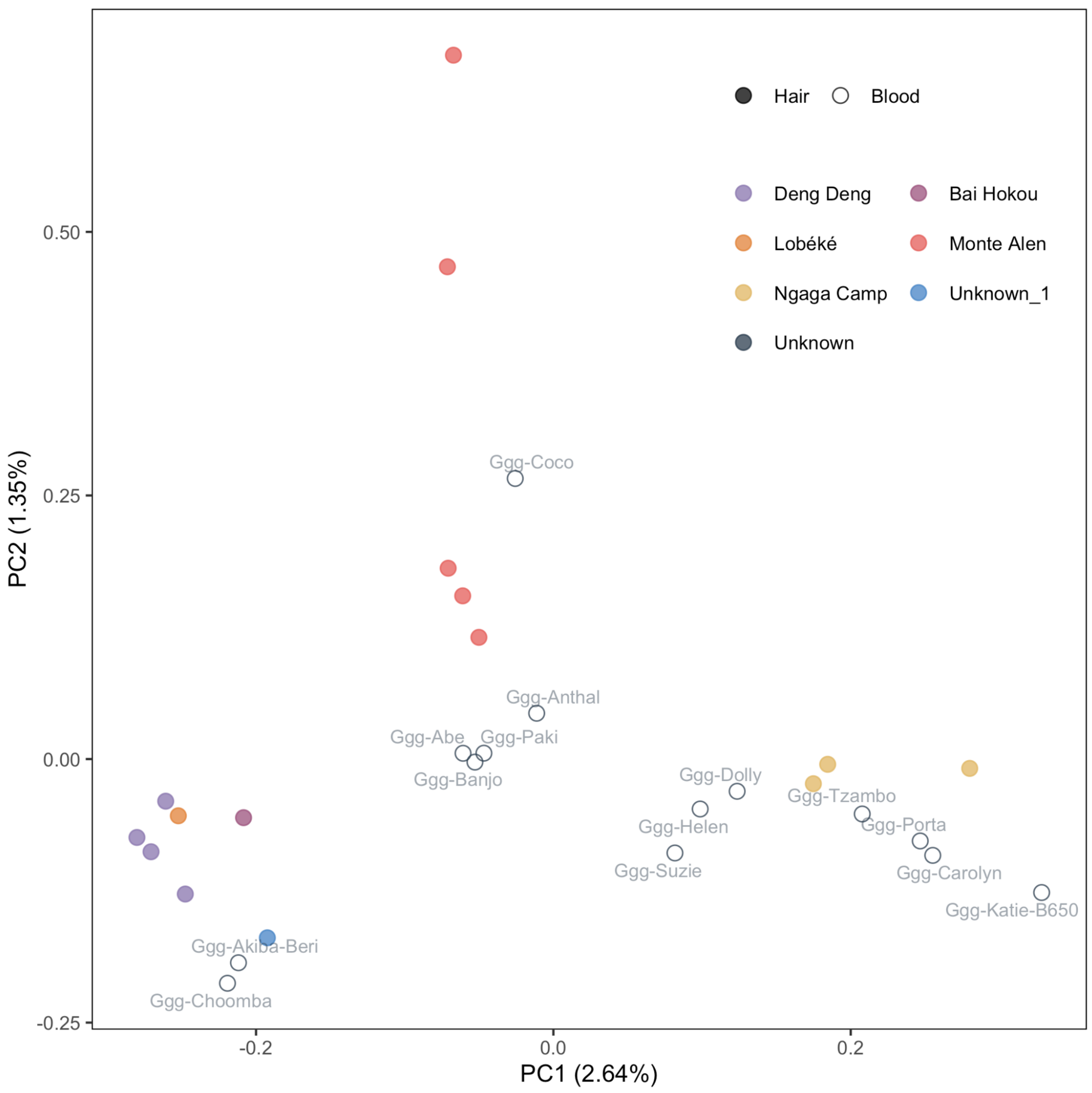

Figure S13. PCA of western lowland gorillas. PCA from PCANGSD using western lowland gorilla (WL) hair samples after quality control and published blood samples (Set03). Color represents each site.

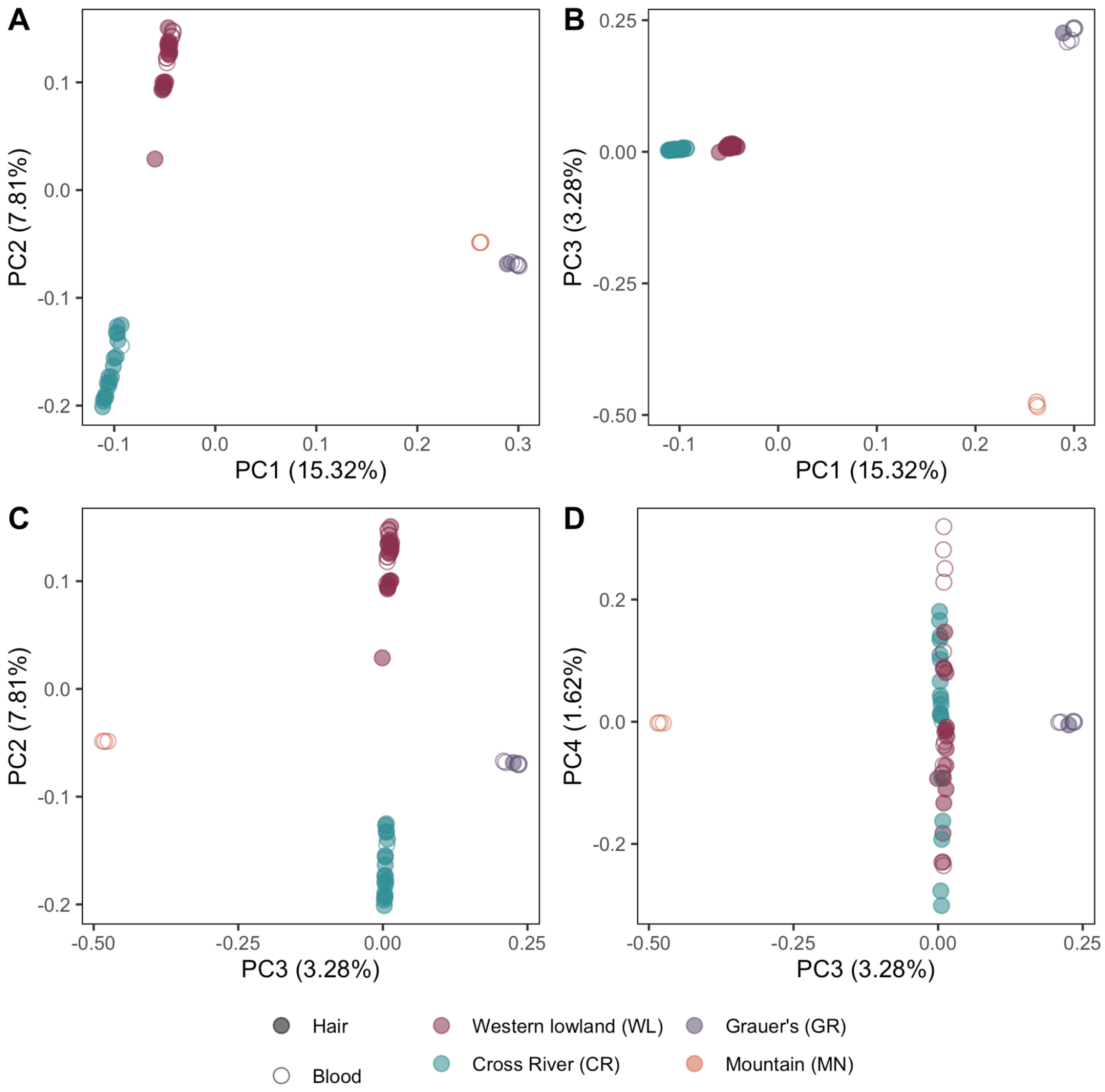

Figure S14. PCA with the 4 gorilla subspecies. PCA from PCANGSD using samples after quality control with final subspecies assignment (Set02). Shapes represent the type of sample, hairs with filled circles and blood with empty circles. Color represents the gorilla subspecies. **A**, PC1 and PC2; **B**, PC1 and PC3; **C**, PC3and PC2 and **D** PC3 and PC4.

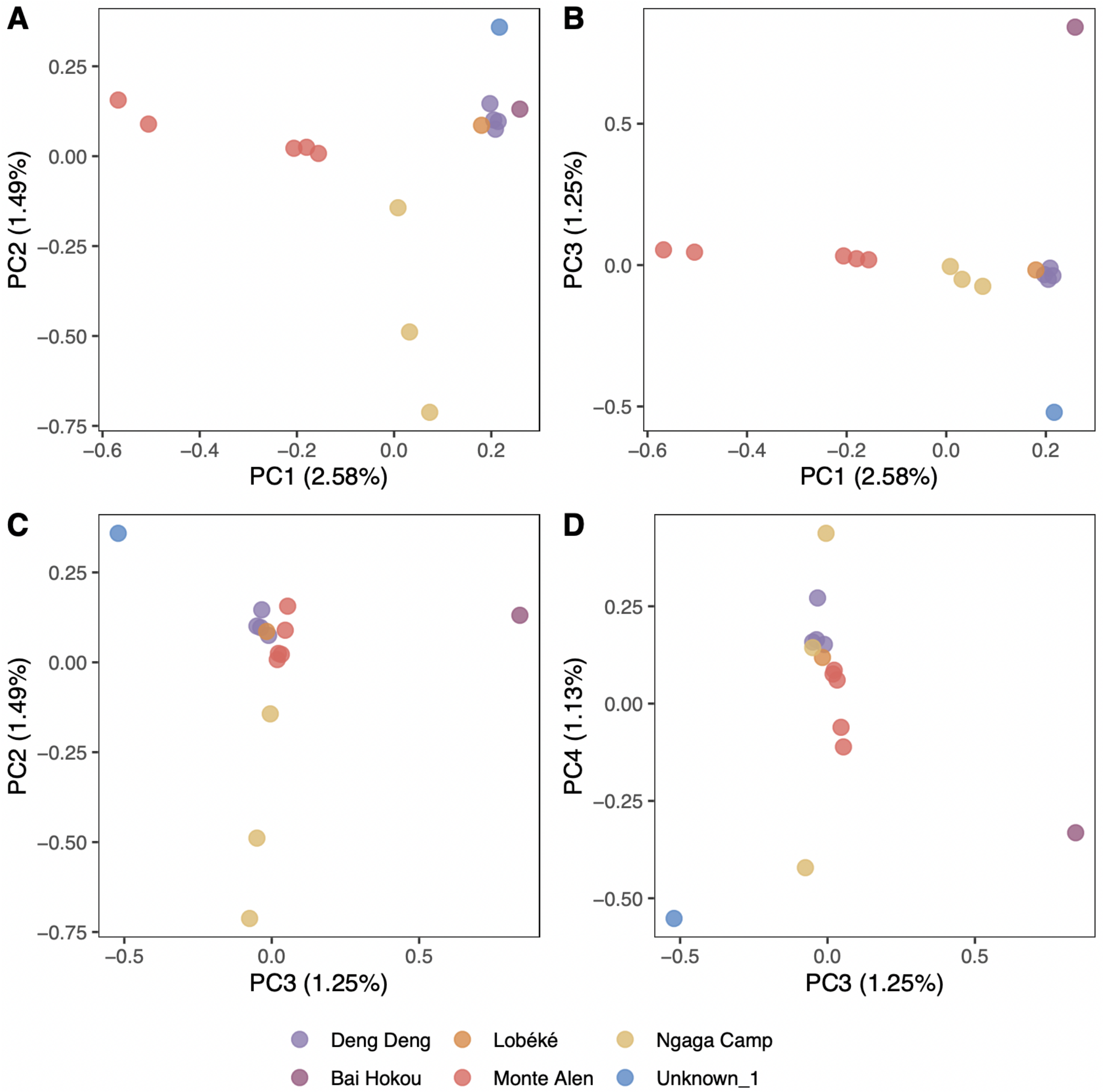

Figure S15. PCA western lowland gorilla hairs. PCA from PCANGSD using western lowland gorilla hair samples after quality control (Set06). Color represents each site. **A**, PC1 and PC2; **B**, PC1 and PC3; **C**, PC3and PC2 and **D** PC3 and PC4.

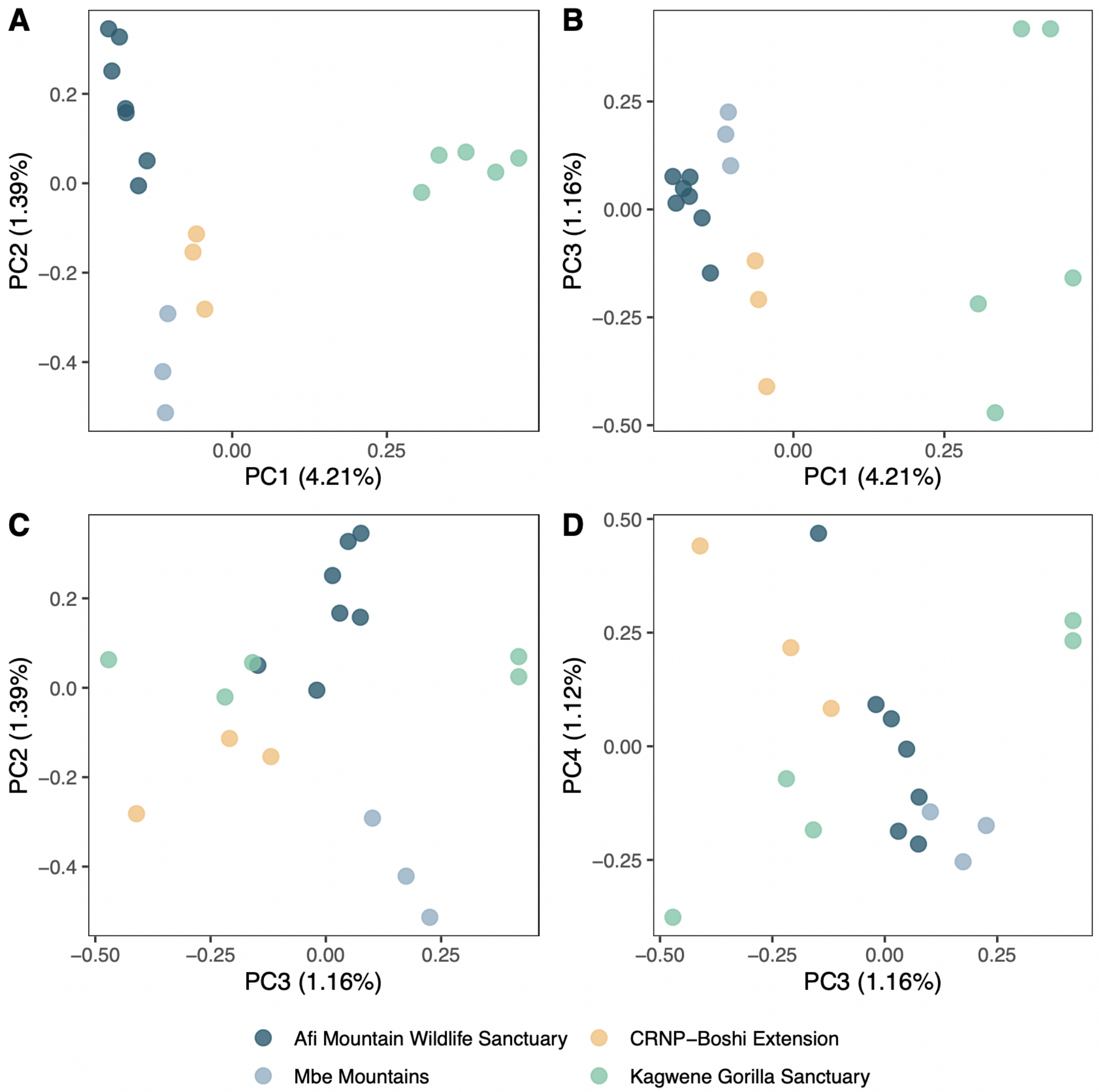

Figure S16. PCA Cross River gorilla hairs. PCA from PCANGSD using Cross River gorilla hair samples after quality control (Set07). Color represents each site. A, PC1 and PC2; B, PC1 and PC3; C, PC3and PC2 and D PC3 and PC4.

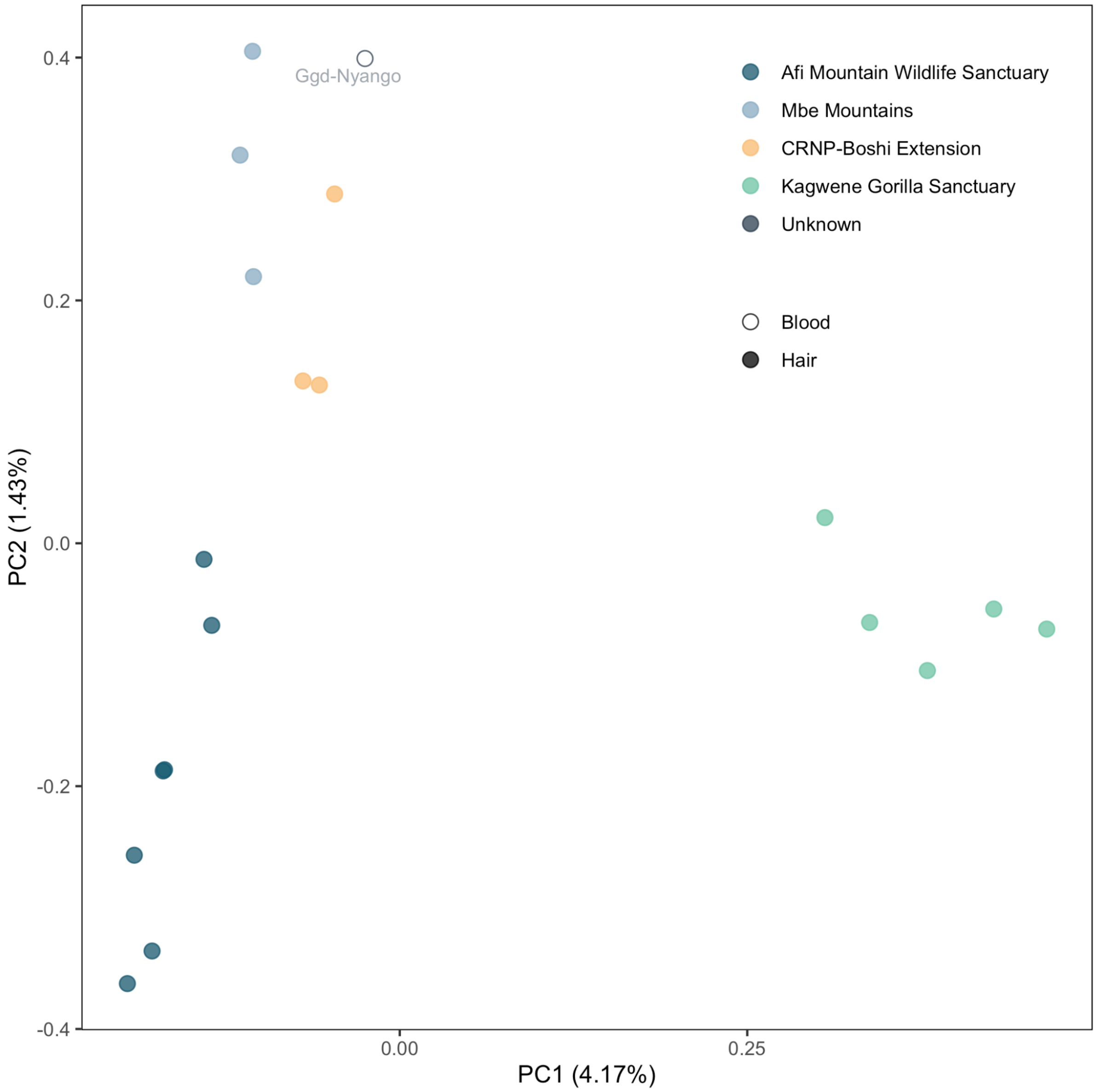

Figure S17. PCA of Cross River gorillas. PCA from PCANGSD using Cross River (CR) gorilla hair samples after quality control and published blood samples (Set04). Color represents each site.

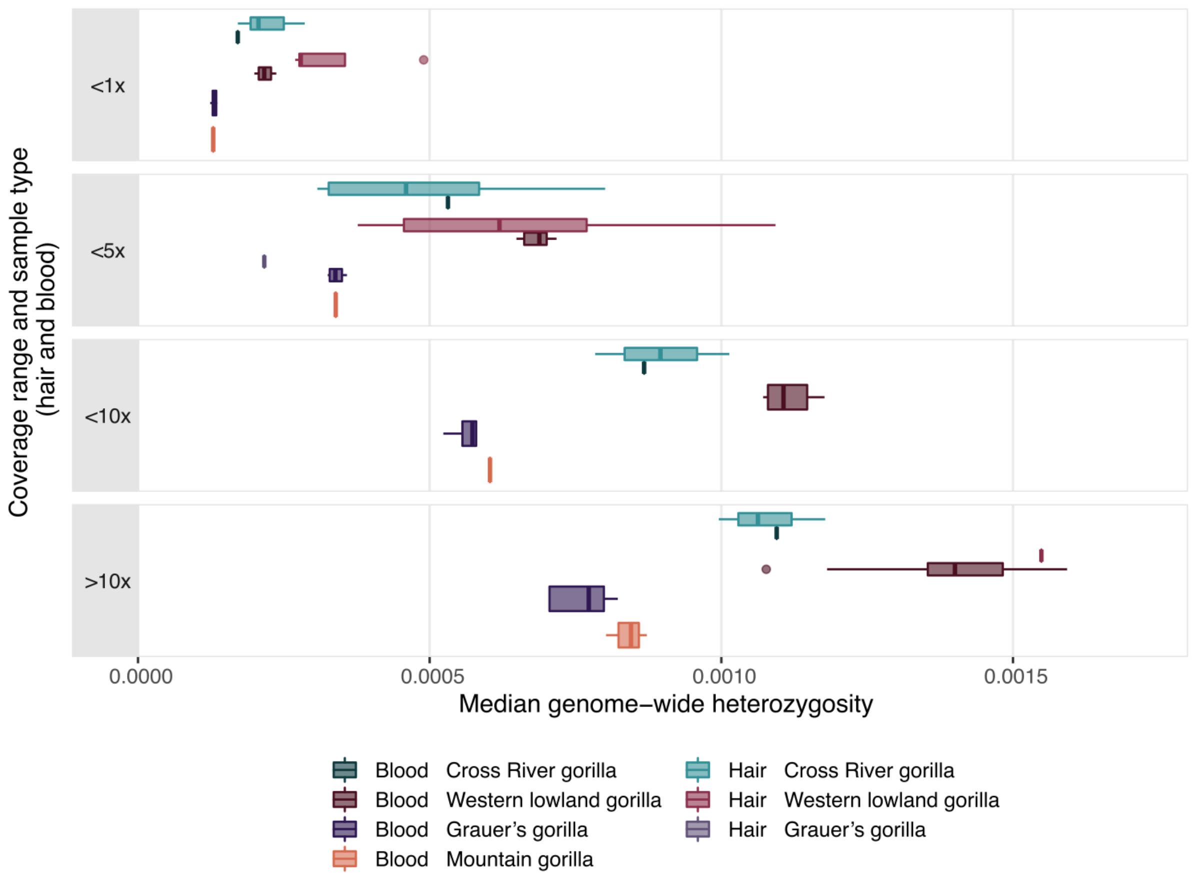

Figure S18. Genome-wide heterozygosity. Genome-wide heterozygosity estimates by sample type and species. Color indicates gorilla subspecies, with darker colors indicating blood samples and lighter indicating hair samples. Blood samples have been downsampled to 0.75x, 3x and 7x to serve as reference in the coverage ranges of <1x, <5x and <10x.

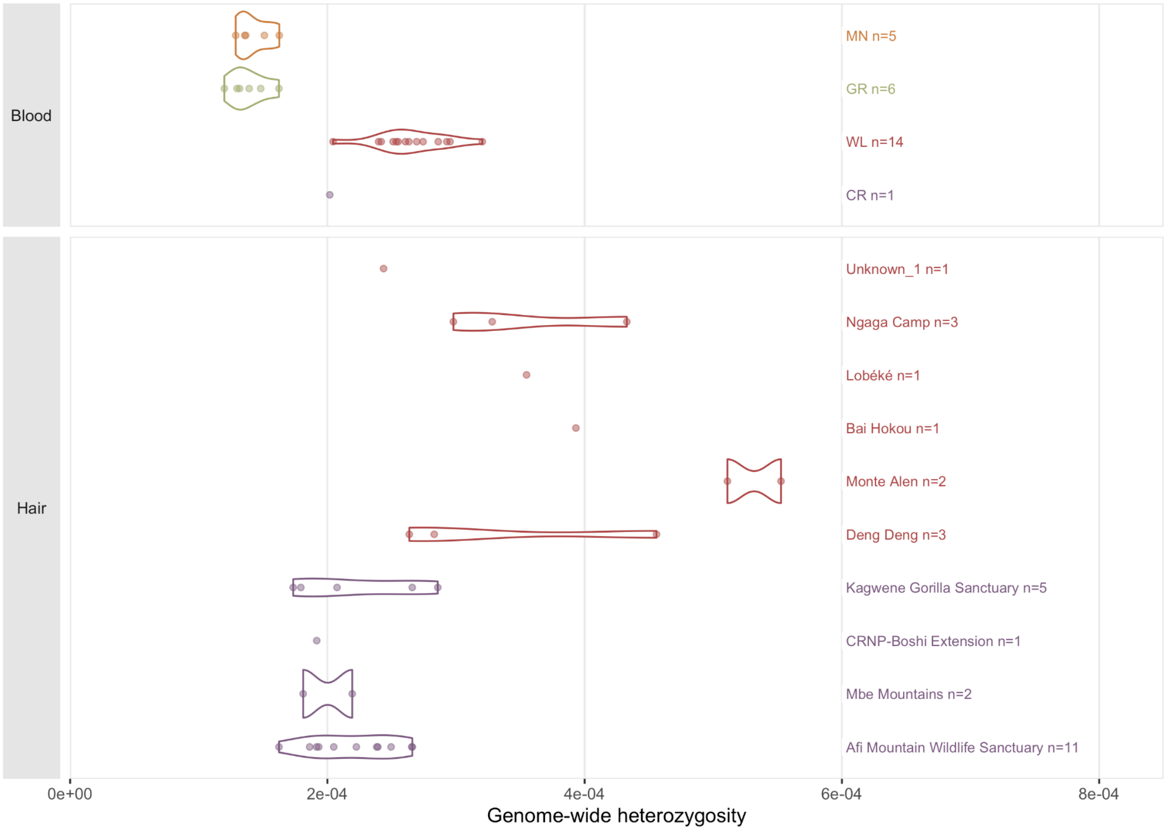

Figure S19. Genome-wide heterozygosity with downsampling. Genome-wide heterozygosity estimated with ANGSD on samples with coverage >0.8x and those with higher coverage than 1.1x which we downsampled to an average of 1x. The color indicates gorilla subspecies, WL in red, CR in green, GR in purple, and MN in orange.

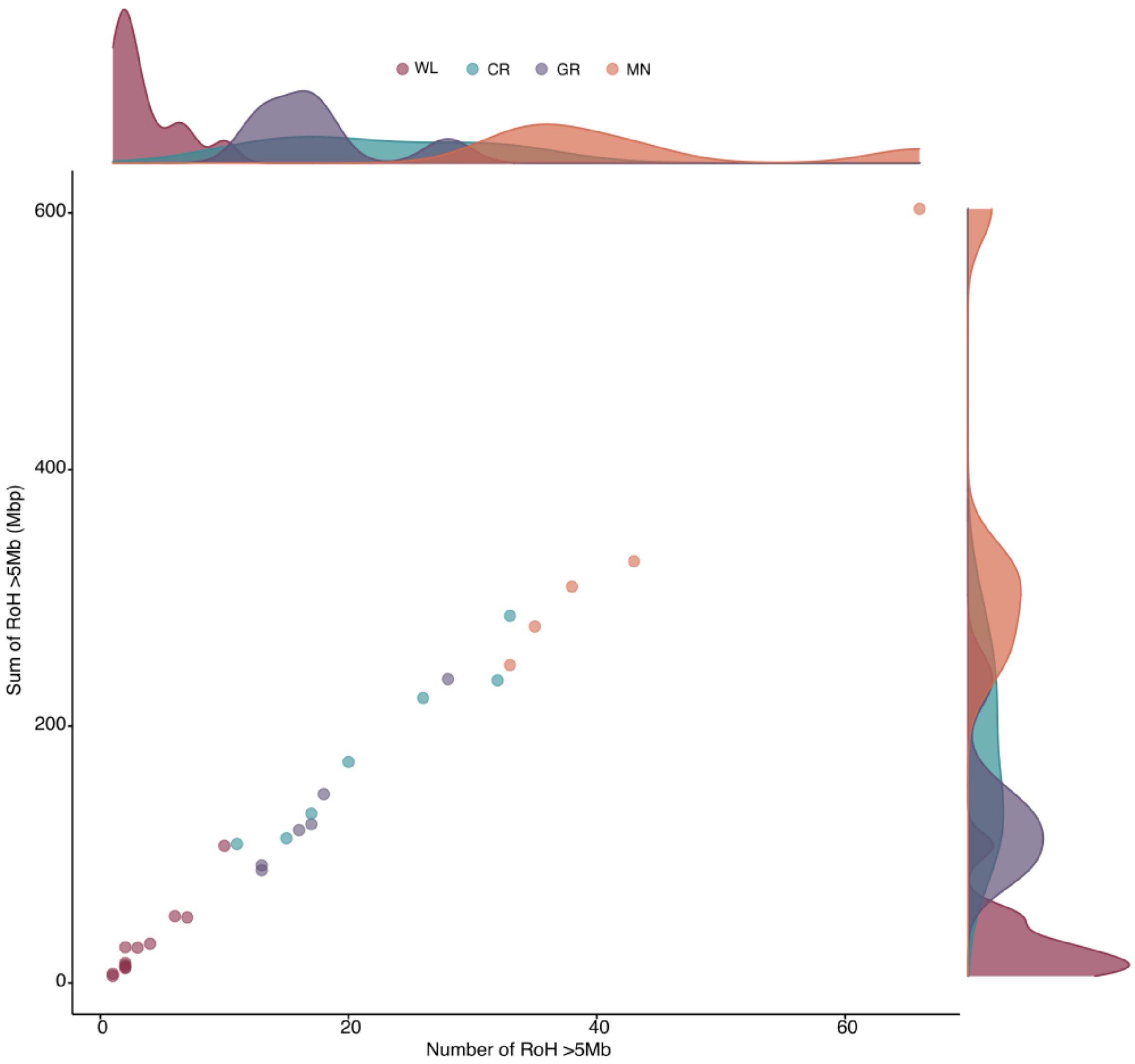

Figure S20. RoH sum and number. Distribution of the sum of RoHs for each species versus count of RoHs, all of size >= 5Mbp. Mountain gorilla (MN) in orange, Grauer's gorilla (GR) in purple, Cross River (CR) gorilla in green and western lowland (WL) gorilla in red.

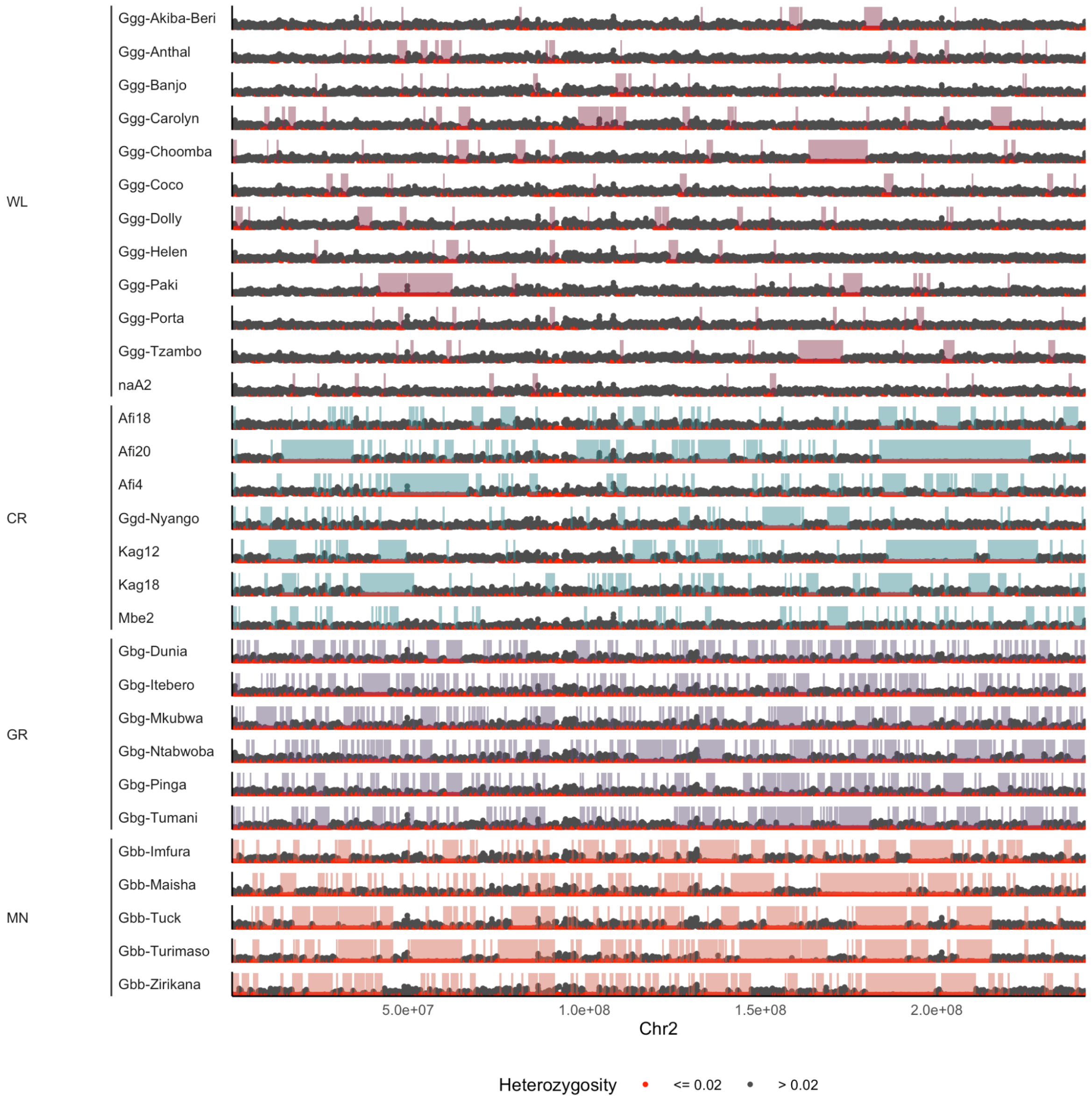

Figure S21. RoH along Chr2. Runs of homozygosity from BCFTools/RoH along chromosome 2 for the high-coverage samples downsampled at an average coverage of 10x. Points represent heterozygosity values estimated in windows of 100kbp with 50kbp overlap. Points color represent heterozygosity below 0.02 in red, and above in black. Segment colors represent species.

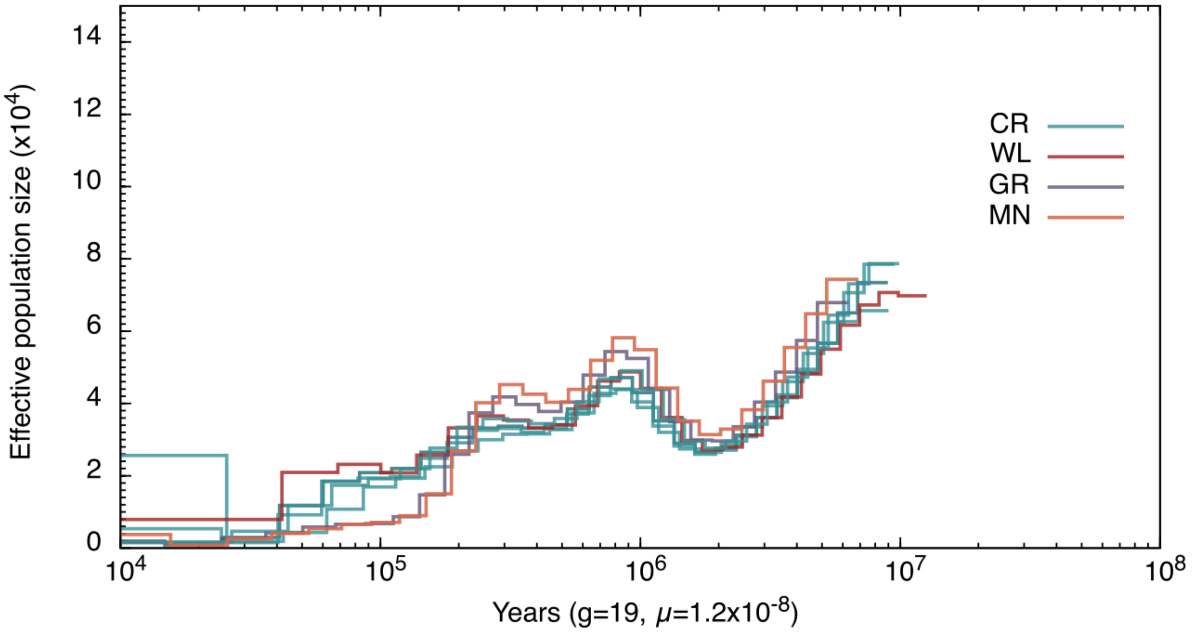

Figure S22. PSMC gorillas. PSMC on high coverage samples (>=7x) for the 4 gorilla subspecies including both hair and blood samples. Generation time set to 19 years and mutation rate to 1.2e^{-8}.

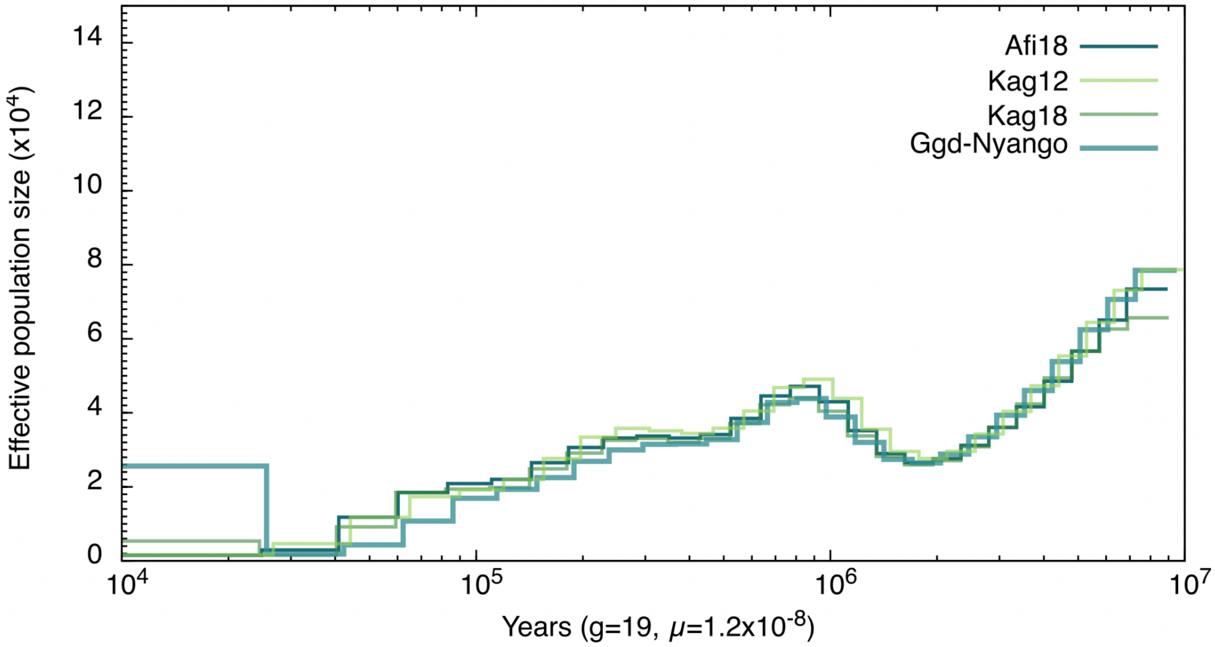

Figure S23. PSMC Cross River gorillas. PSMC on high coverage samples (<7x) for the Cross River gorillas, including both hair and blood samples. Ggd-Nyango is a blood sample from Prado-Martinez et al. 2013 and the rest are hair samples from this study. Generation time set to 19 years and mutation rate to 1.2e^{-8}.

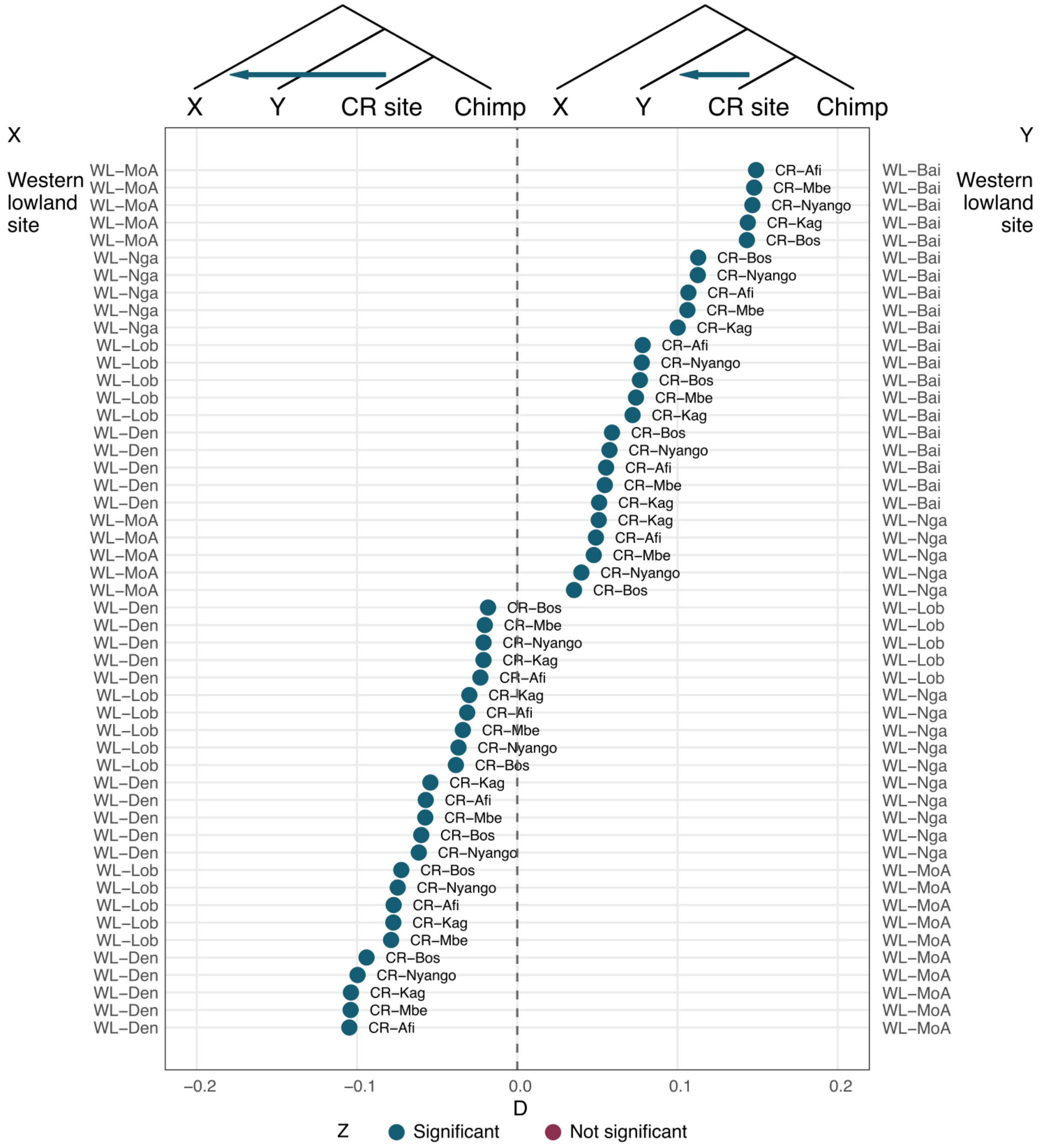

Figure S24. *D* statistics test (ABBA-BABA) between western populations calculated with ANGSD. Color represents the Z score: blue is significant (<= -3 or >= 3) and red, is not significant. Positive values indicate that Cross River gorillas (CR) have greater affinity with the western lowland gorilla (WL) population Y (right Y axis labels); negative values indicate greater affinity with WL population X (left Y axis labels).

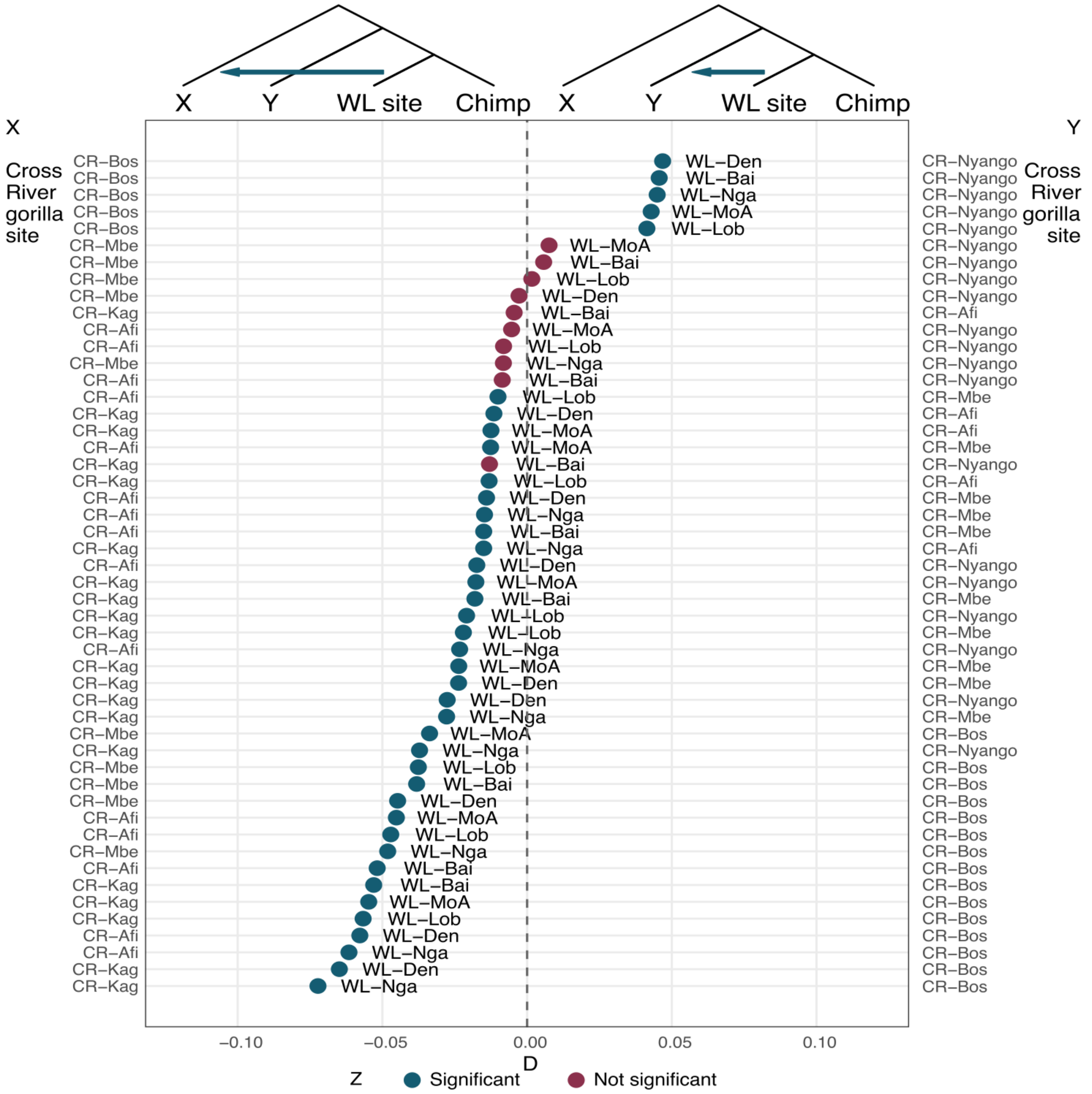

Figure S25. *D* statistics test (ABBA-BABA) between western populations calculated with ANGSD. Color represents Z score: blue is significant (<= -3 or >= 3) and red, not significant. Positive values indicate that western lowland gorillas (WL) have greater affinity with the CR population Y (right Y axis labels); negative indicate greater affinity with WL population X (left Y axis labels).

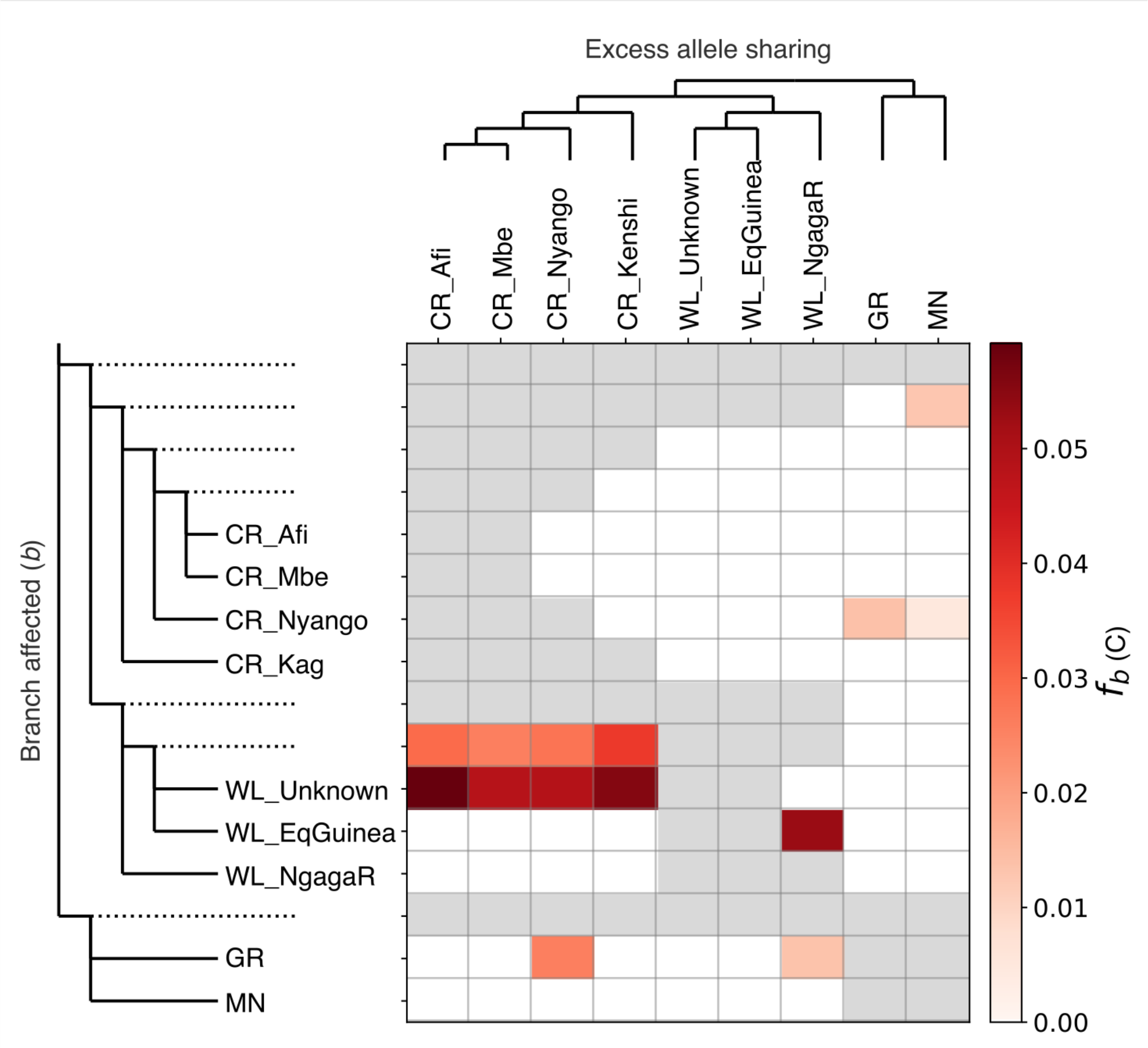

Figure S26. *F*-branch CR sites, WL regions and eastern subspecies. *F-branch* (f_b(C)) analysis on excess allele sharing between Eastern gorilla species, CR sites/regions and WL regions from PCA clustering (using high coverage hair samples and reference blood samples downsampled to an average coverage of 10x. The x-axis and y-axs trees are the species tree and the expanded species trees (with internal branches indicated with dotted lines), respectively. Grey-coloured cells indicate comparisons for which gene flow could not be inferred based on the given tree topology. Red color indicates f_b value; darker color indicates greater allele sharing between the expanded tree branch (relative to its sister branch) (*b*) and the populations on the x-axis (C).

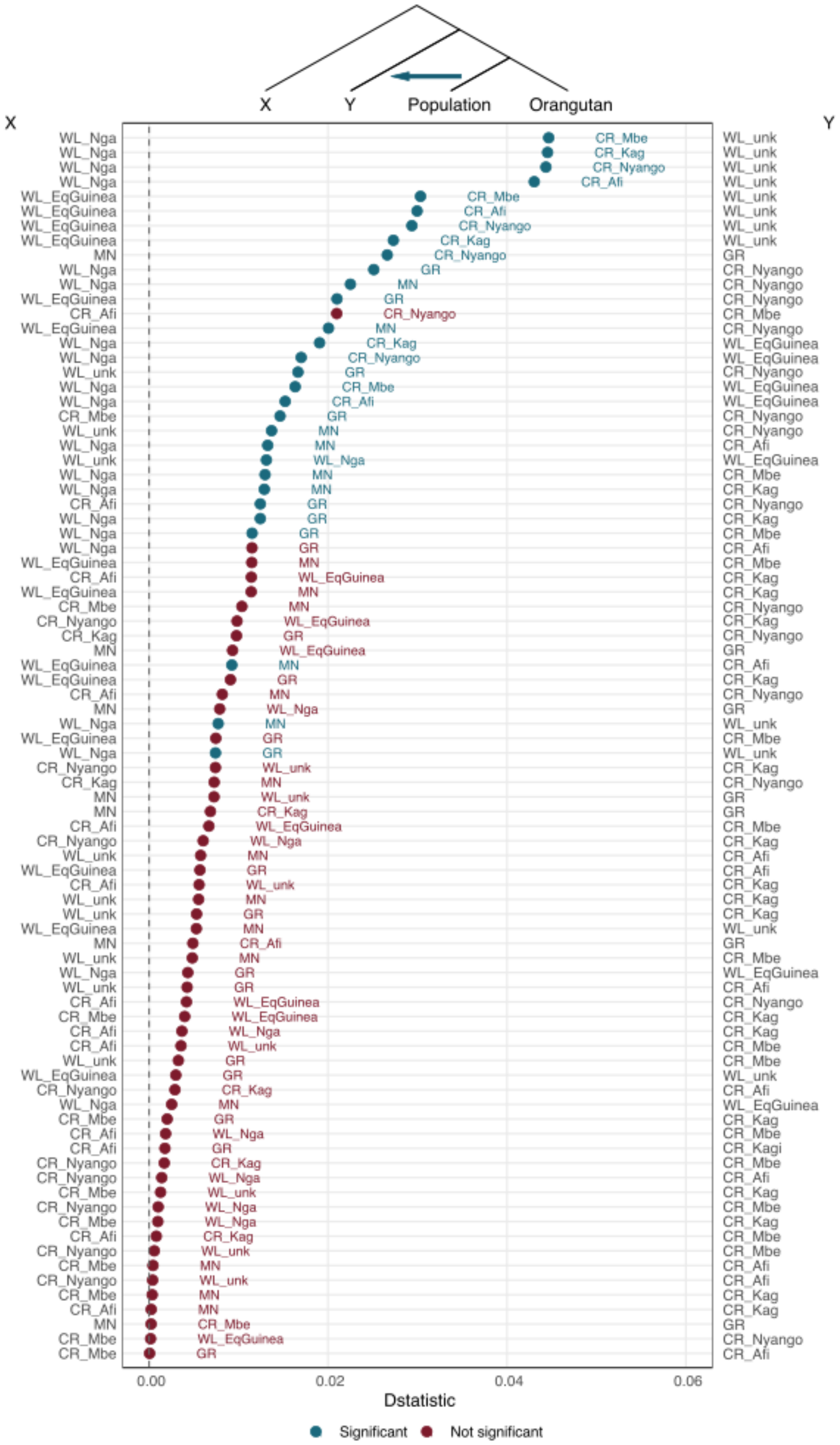

Figure S27. *D*stats CR sites, WL regions. *D* statistics test (ABBA-BABA) between Cross River gorilla (CR) sites, western lowland gorilla (WL) regions and eastern gorilla subspecies calculated with Dsuite. Color represents Z score: blue is significant (<= -3 or >= 3) and red, not significant. Positive values indicate that the population labeled has greater affinity with the population Y (right Y axis labels); negative indicate greater affinity with population X (left Y axis labels).

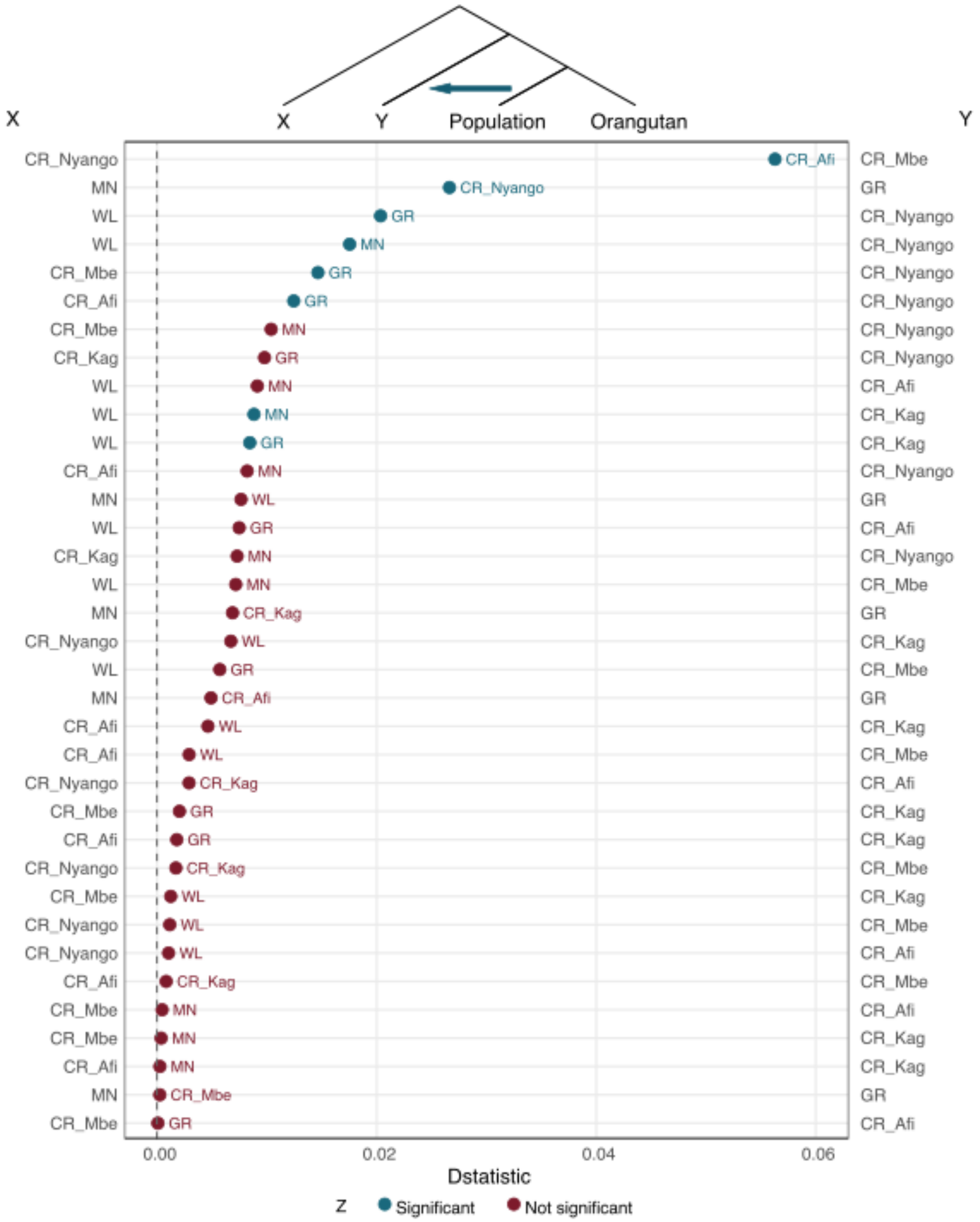

Figure S28. *D*stats CR sites. *D* statistics test (ABBA-BABA) between Cross River sites and the rest of gorilla subspecies calculated with Dsuite. Color represents Z score: blue is significant (<= -3 or >= 3) and red, not significant. Positive values indicate that the population labeled has greater affinity with the population Y (right Y axis labels); negative indicate greater affinity with population X (left Y axis labels).

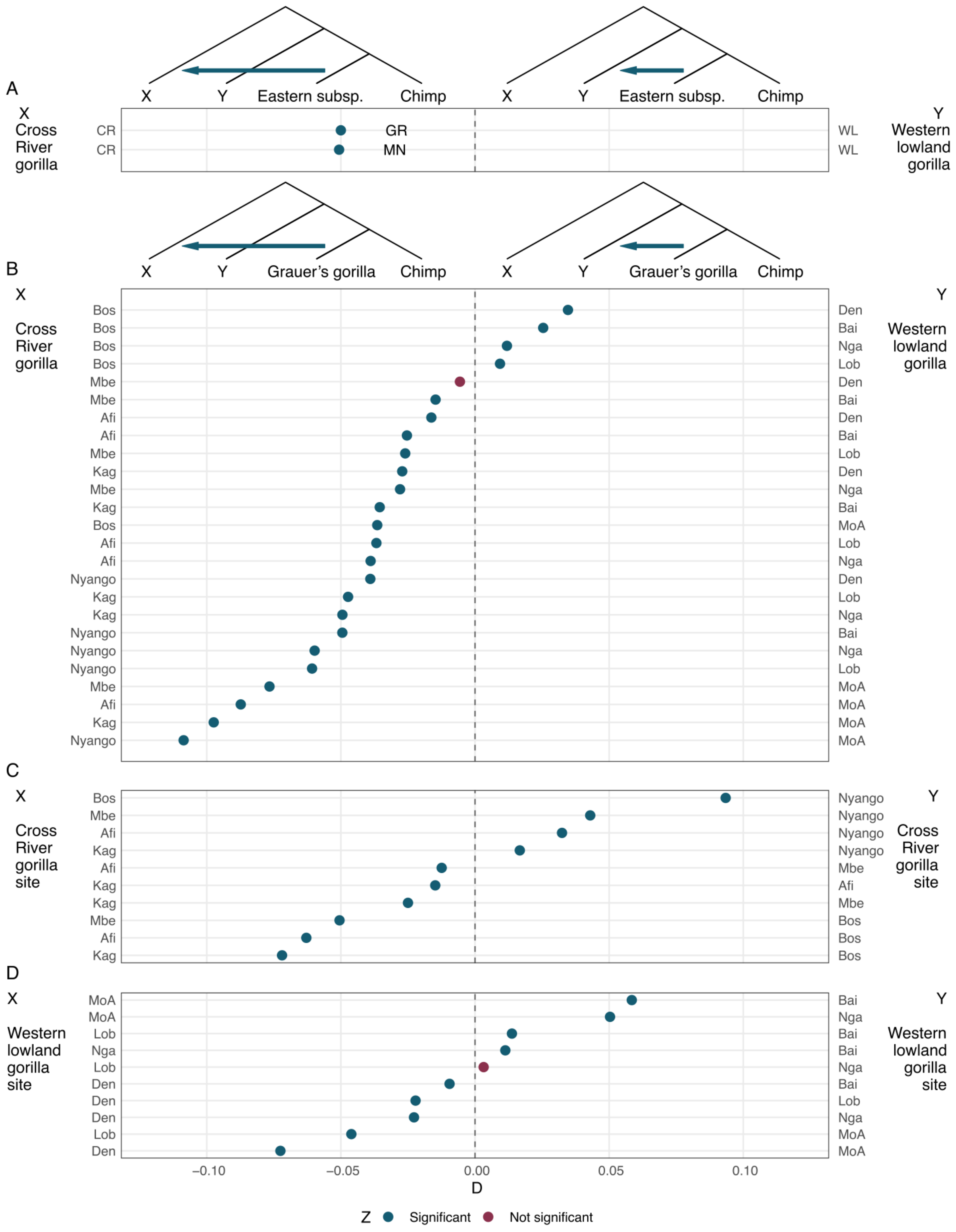

Figure S29. *D*stats populations. *D* statistics test (ABBA-BABA) between populations (CR and WL) calculated with ANGSD. Color represents Z score, blue is significant (<= -3 or >= 3) and red not significant. Positive values indicate that eastern gorillas (Grauer's gorillas, GR & Mountain gorillas, MN) (panel A) or Grauer's (GR) (B, C, D panel) have greater affinity with species or population Y (right Y axis labels); negative indicate greater affinity with species or population X (left Y axis labels).

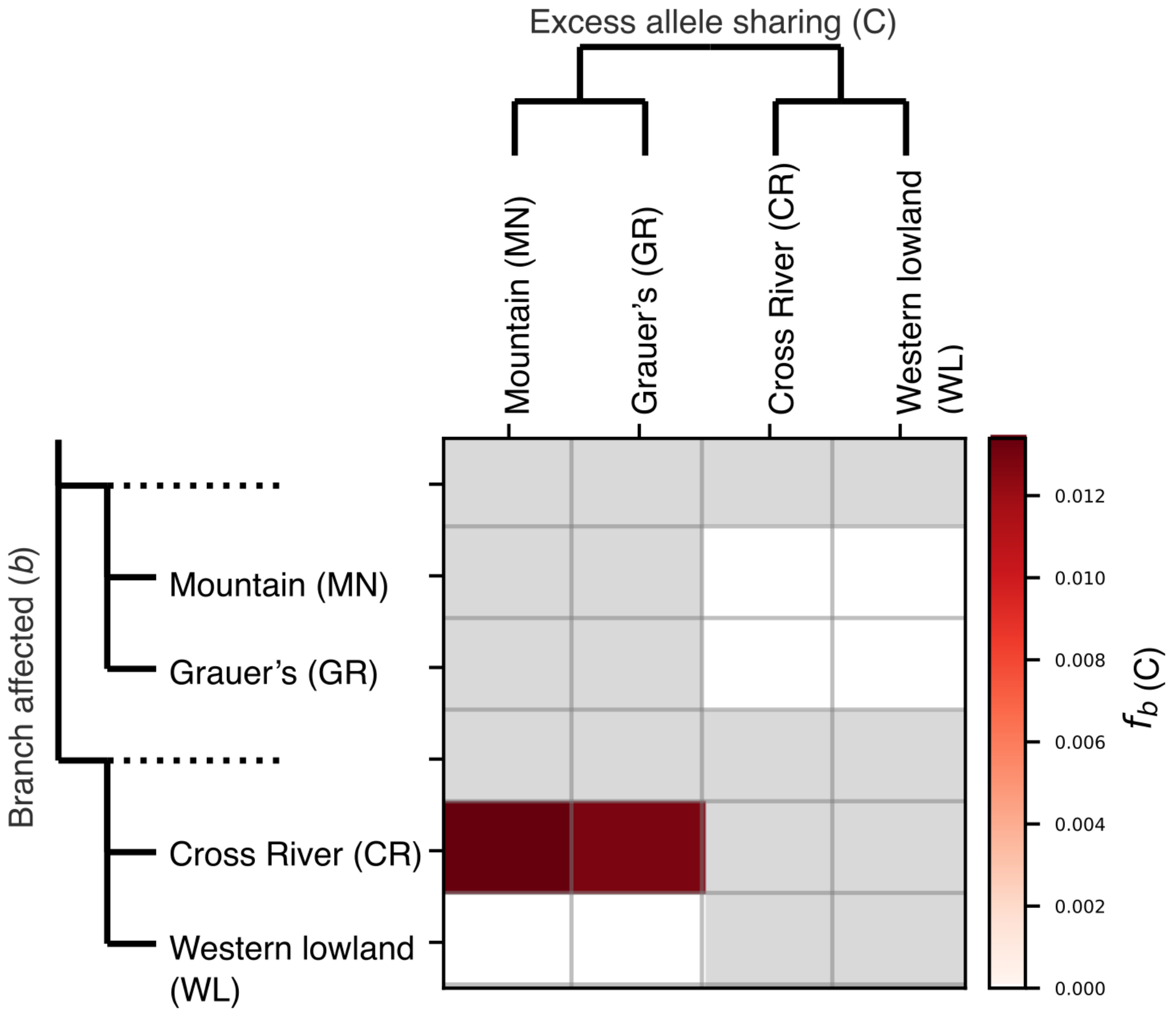

Figure S30. *F-branch* species. *F-branch* (f_b(C)) analysis on excess allele sharing between gorilla species (using high coverage hair samples and reference blood samples downsampled to an average coverage of 10x. The x-axis and y-axs trees are the species tree and the expanded species trees (with internal branches indicated with dotted lines), respectively. Grey-coloured cells indicate comparisons for which gene flow could not be inferred based on the given tree topology. Red color indicates f_b value; darker color indicates greater allele sharing between the expanded tree branch (relative to its sister branch) (*b*) and the populations on the x-axis (C).

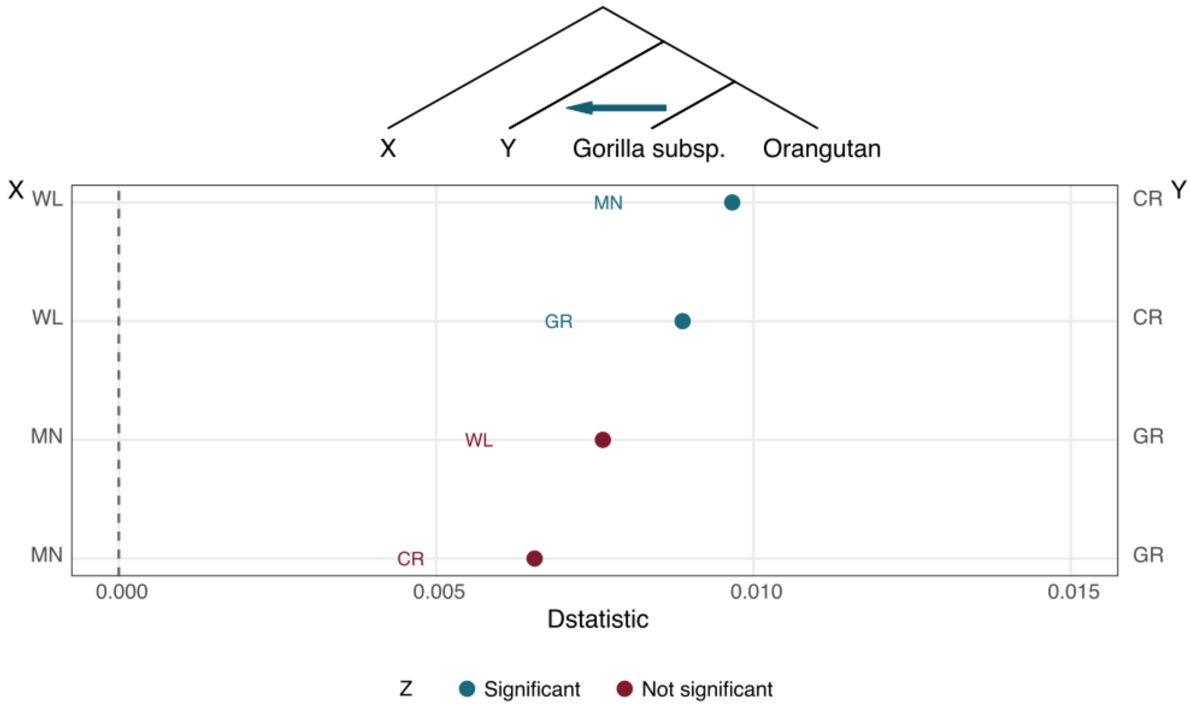

Figure S31. *D*stats gorilla subspecies. *D* statistics test (ABBA-BABA) between gorilla subspecies calculated with Dsuite. Color represents Z score, blue is significant (<= -3 or >= 3) and red not significant. Positive values indicate that the corresponding gorilla subspecies has greater affinity with subspecies Y (right Y axis labels); negative indicate greater affinity with subspecies X (left Y axis labels).

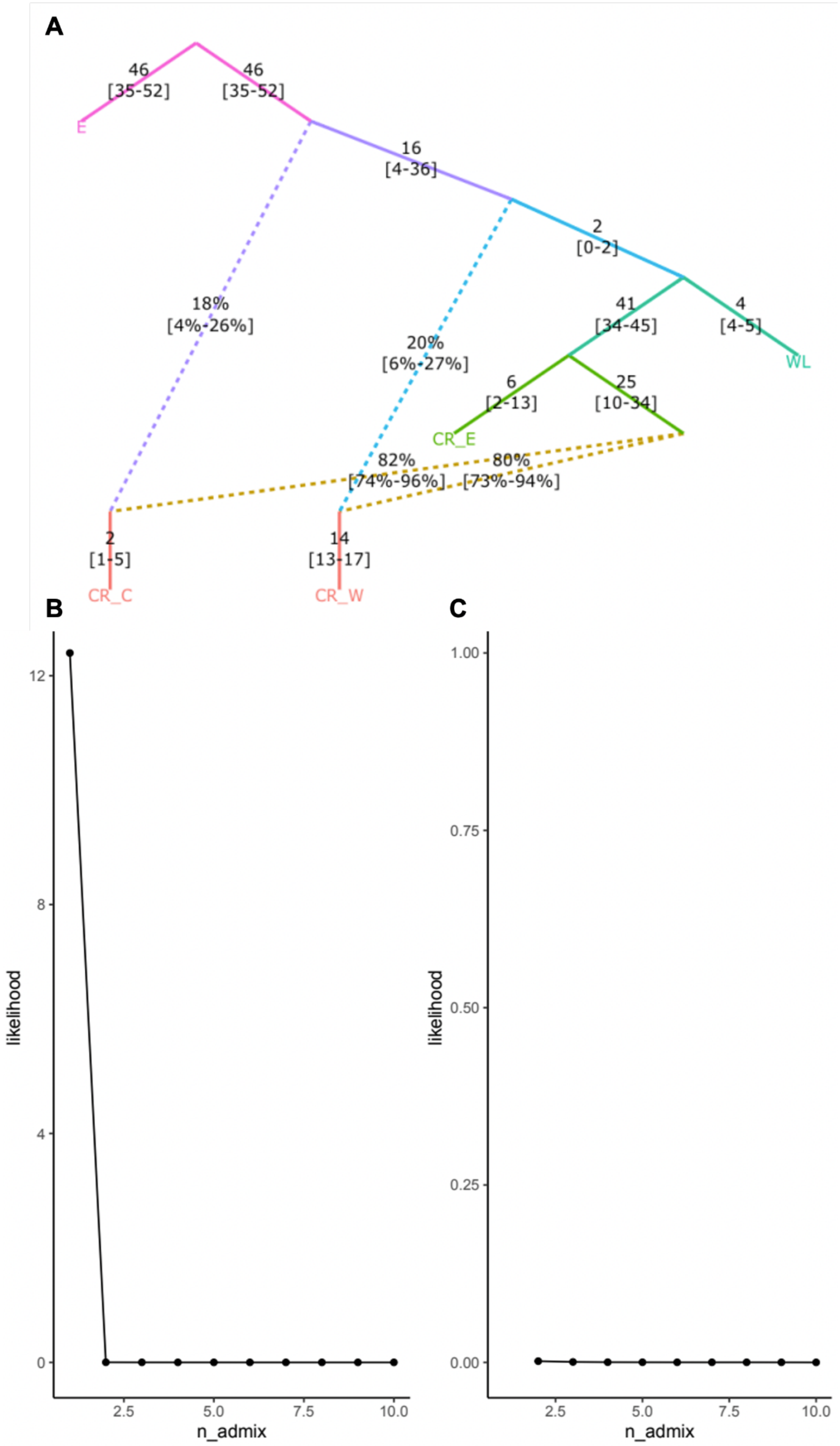

Figure S32. Admixturegraph. Admixturegraph considering five populations (CR-west, CR-central, CR-east, western lowland (WL), and eastern gorillas as an outgroup). **B, C.** qpgraph likelihood scores.

Supplementary Tables

Table S1. Excel spreadsheet with metadata.

Table S2. Final dataset. The number of samples by species and site that pass quality control (QC) filters (had an estimation below 1% of putative human contamination from the HuConTest and coverage >= 0.5x). The second and third columns show the number of samples that we discard/kept when taking into account relatedness test results for several analyses.

	Keept after relatedness test		
**Species/Site**	**No**	**Yes**	**Total**
**Cross River gorilla**			
Afi Mountain Wildlife Sanctuary	6	7	13
CRNP-Boshi Extension	0	3	3
Kagwene Gorilla Sanctuary	1	5	6
Mbe Mountains	0	3	3
**Grauer's gorilla**			
Unknown_3	0	1	1
**Western lowland gorilla**			
Bai Hokou	0	1	1
Deng Deng	0	4	4
Lobéké	0	1	1
Monte Alen	0	5	5
Ngaga Camp	0	3	3
Unknown_1	0	1	1

Table S3. Fst values.

	Afi	Mbe	Bos	Kag	Den	MoA	Nga
Afi	0.000000	0.096871	0.086526	0.169374	0.236934	0.306053	0.268134
Mbe	0.096871	0.000000	0.112591	0.198408	0.262504	0.325810	0.288888
Bos	0.086526	0.112591	0.000000	0.171279	0.230014	0.293078	0.261068
Kag	0.169374	0.198408	0.171279	0.000000	0.229102	0.300341	0.260372
Den	0.236934	0.262504	0.230014	0.229102	0.000000	0.158964	0.117682
MoA	0.306053	0.325810	0.293078	0.300341	0.158964	0.000000	0.162068
Nga	0.268134	0.288888	0.261068	0.260372	0.117682	0.162068	0.000000
